# Targeting LHPP in neoadjuvant chemotherapy resistance of gastric cancer: insights from single-cell and multi-omics data on tumor immune microenvironment and stemness characteristics

**DOI:** 10.1038/s41419-025-07614-z

**Published:** 2025-04-16

**Authors:** You-Xin Gao, Xiao-Jing Guo, Bin Lin, Xiao-Bo Huang, Ru-Hong Tu, Mi Lin, Long-Long Cao, Qi-Yue Chen, Jia-Bin Wang, Jian-Wei Xie, Ping Li, Chao-Hui Zheng, Ying-Hong Yang, Chang-Ming Huang, Jian-Xian Lin

**Affiliations:** 1 Department of Gastric Surgery, Fujian Medical University Union Hospital, Fuzhou, China; 2 Key Laboratory of Ministry of Education of Gastrointestinal Cancer, Fujian Medical University, Fuzhou, China; 3 Fujian Key Laboratory of Tumour Microbiology, Fujian Medical University, Fuzhou, China; 4 Department of Pathology, Fujian Medical University Union Hospital, Fuzhou, China; 5 Gastrointestinal Cancer Institute, Fujian Medical University, Fuzhou, 350001 China

**Keywords:** Cancer microenvironment, Cancer stem cells

## Abstract

Gastric cancer (GC) is a highly heterogeneous and complex malignancy, often characterized by tumor stemness and immune evasion mechanisms, which contribute to a poor response to neoadjuvant chemotherapy (NAC) and treatment resistance. In this study, we performed a comprehensive analysis using single-cell and multi-omics approaches on 375 GC samples from The Cancer Genome Atlas database, along with 141 clinical samples from patients who underwent NAC. We identified key gene modules associated with stemness and immune traits, and developed a novel stem cell-immune risk score. This score effectively distinguished responders from non-responders to chemotherapy, and was significantly associated with overall survival. Through multi-omics analysis, we further elucidated the role of phospholysine phosphohistidine inorganic pyrophosphatase (*LHPP*) in the tumor immune microenvironment. Our findings showed that high *LHPP* expression was closely linked to the increased infiltration of antitumor immune cells, such as CD8^+^ T cells, and significantly suppressed the development of stemness characteristics in GC. Additionally, single-cell sequencing data revealed that tumor epithelial cells with low *LHPP* expression exhibited heightened stemness and demonstrated the strongest communication with CD8^+^-exhausted T cells. We also observed that *LHPP* inhibited stemness and chemotherapy resistance in GC cells by regulating the phosphorylation of GSK-3β. In conclusion, LHPP plays a critical regulatory role in the stemness features and tumor immune microenvironment of GC, presenting a promising biomarker and potential therapeutic target for personalized treatment of GC.

## Introduction

Gastric cancer (GC) is the fifth most common cancer worldwide and the third leading cause of cancer-related mortality [1]. Owing to the nonspecific clinical symptoms and insidious onset of GC, most patients are diagnosed at an advanced stage, with a 5-year survival rate below 40% post-treatment [2, 3]. In early stage patients, the 5-year survival rate can reach 90%. The primary causes of death in patients with advanced GC are tumor invasion and metastasis, which are significant barriers to treatment efficacy and pose severe threats to patient health and life expectancy [4]. The standard treatment for advanced GC is multi-agent chemotherapy; however, most patients develop chemoresistance, with a median overall survival of less than 1 year [5].

Currently, postoperative adjuvant chemotherapy is an essential therapeutic approach for reducing the risk of recurrence in patients with GC [6, 7]. Chemotherapy can directly kill tumor cells or increase their susceptibility to immune effects, thereby enhancing tumoricidal activity [8, 9]; however, chemoresistance limits its clinical application. Chemoresistance arises through clonal evolution of tumor cells, with multiple cell populations derived from initial tumor-initiating cells known as cancer stem cells (CSCs) [10]. Research by Pan et al. [11] indicated that CSCs are more resistant to chemotherapy than non-CSCs, and may result in cancer metastasis. In recent years, immunotherapy has shown significant antitumor effects in the treatment of various solid tumors. Immunotherapy enhances the antitumor effects of the immune system by reactivating the suppressed immune microenvironment, and has become a first-line treatment for certain tumors [12, 13]. However, the response rate of GC to immunotherapy is low, which may be related to the presence of stem cells within the tumor [14]. CSCs promote tumor-associated immune and stromal cells to adopt a pro-tumorigenic phenotype through paracrine and juxtacrine signaling, leading to resistance to immunotherapy [15].

Phospholysine phosphohistidine inorganic pyrophosphatase (*LHPP*), a histidine phosphatase, plays a broad role in protein and cellular functions, including cell cycle regulation, phagocytosis, and modulation of ion channel activity, among others [16, 17]. It is widely expressed in various cancer tissues [18] and exhibits tumor-suppressive effects in multiple cancers, including liver [19, 20], gastric [21], colorectal [22], and pancreatic [23] cancers. *LHPP* functions as a tumor suppressor gene in GC by regulating the PI3K/AKT/mTOR pathway [24] and inhibiting the Wnt/β-catenin signaling pathway [25], the TGFβ/Smad signaling pathway [26], AKT phosphorylation [27], and other mechanisms to suppress tumor occurrence and progression. Previous studies have shown that low expression of *LHPP* is significantly correlated with poor prognosis and chemosensitivity in patients with GC and that *LHPP* is a potential predictive biomarker and therapeutic target for GC [21]. Therefore, this study aimed to comprehensively investigate the mechanisms of drug resistance in GC using multi-omics and diverse computational methods, potentially providing new biomarkers for predicting the prognosis of patients with GC and personalized treatment plans.

## Result

### Classification and identification of stem cell characteristics and immune infiltration features in patients with GC

**Fig. 1:**
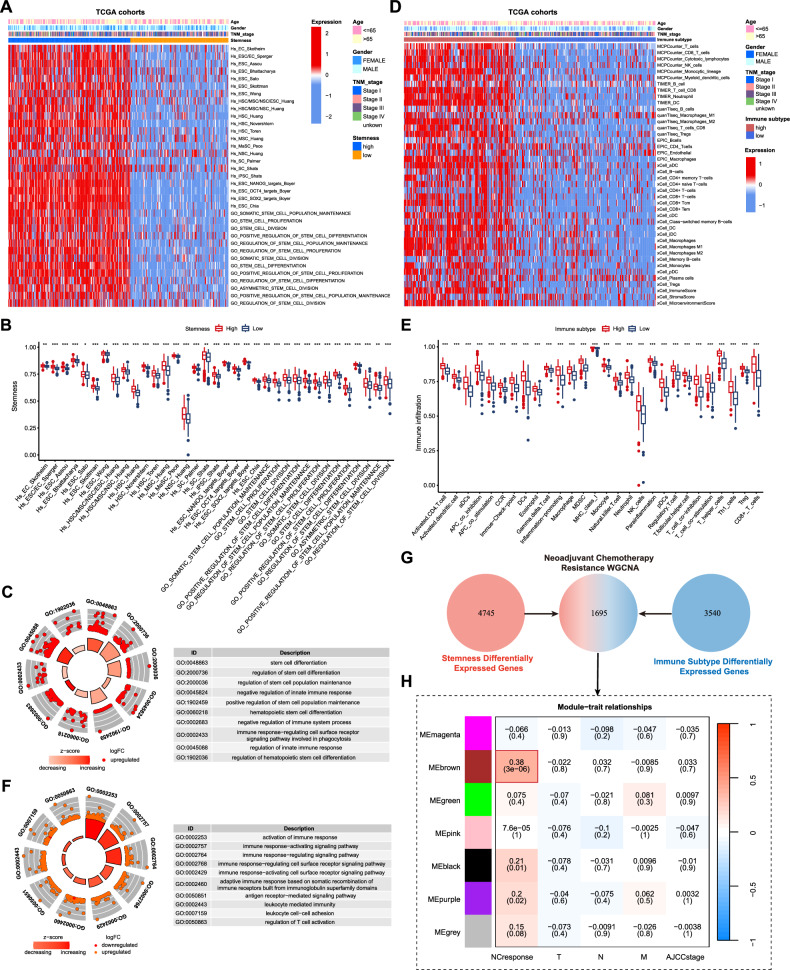
Integrative analysis of stemness and immune characteristics in the transcriptome of gastric cancer patients. **A** The integrated heatmap illustrates the stemness pathway scores for each patient across the two stemness phenotypes. **B** Student’s *t* test was used to evaluate the differences in stemness pathway scores between the two stemness subgroups. The upper and lower edges of the box represent the interquartile range, the line within the box denotes the median, and the dots indicate outliers. **C** GO enrichment analysis illustrated the pathway enrichment profiles within the two stemness subgroups. **D** The integrated heatmap displays the immune cell infiltration profiles for each patient across the two immune phenotypes. **E** Student’s *t* test was used to assess differences in immune cell infiltration between the two immune subgroups. The upper and lower edges of the box represent the interquartile range, the line within the box indicates the median, and the dots denote outliers. **F** GO enrichment analysis revealed pathway enrichment profiles across the two immune subgroups. **G** Venn diagram illustrates the overlap between differentially expressed genes associated with the stemness subgroups and those associated with the immune subgroups. The intersecting differentially expressed genes were further analyzed using Weighted Gene Co-Expression Network Analysis (WGCNA). **H** The Module–Trait Relationships diagram illustrates the correlations between different modules and the clinical information of neoadjuvant-treated patients. *p < 0.05; **p < 0.01; ***p < 0.001.

This study collected mRNA transcriptome sequencing data from 375 GC samples sourced from The Cancer Genome Atlas (TCGA) database. Subsequently, 35 stemness-related gene sets were identified using the StemChecker tool (http://stemchecker.sysbiolab.eu/) and the Gene Ontology (GO) Consortium (Fig. 1A). To characterize the stem cell-like properties of The Cancer Genome Atlas Stomach Adenocarcinoma cohort, single-sample gene set enrichment analysis (ssGSEA) was employed to quantitatively analyze the enrichment of 35 stemness-related gene sets (Fig. 1A). Subsequently, an unsupervised consensus clustering algorithm was used to classify the patients with stomach adenocarcinoma (STAD) into two distinct immune subgroups (Fig. S1A). Based on the varying stemness enrichment scores calculated using ssGSEA, we defined these subgroups as ‘High Stemness’ and ‘Low Stemness’. The mRNAsi scores showed a significant disparity between the two stemness groups (Fig. S1B). By including the differentially expressed genes significantly over-expressed in the ‘High Stemness’ group, GO enrichment analysis found that, in addition to significant enrichment of stemness-related pathways, a range of negative immune regulation pathways were also enriched in this group (Fig. 1C). SsGSEA indicated significant differences in the composition of the tumor immune microenvironment between different levels of stemness (Fig. S1C). In light of the association between stem cell-like characteristics in GC and the tumor immune microenvironment, we further assessed the abundance of immune cell infiltration in GC using the MCPcounter, QuanTIseq, TIMER, and Xcell tools (Fig. 1D). Using an unsupervised consensus clustering algorithm, we classified patients with STAD into ‘High Immune Subtype’ and ‘Low Immune Subtype’ groups (Figs. 1D and S2A). The reliability of the immune classification in distinguishing patients with differing levels of immune response was further validated through ssGSEA and GO enrichment analyses of genes overexpressed in the ‘High Immune Subtype’ group (Fig. 1E, F). Similarly, we observed distinct enrichment patterns of stemness pathways in the two immune groups, suggesting a robust correlation between stemness and immune characteristics of GC (Figs. S2B and S3A). In addition, we included an analysis of the GEO GC database (GSE15459) and sequencing data from our center to classify and identify GC stem cell characteristics and immune infiltration features, validating the results found in the TCGA database (Figs. S3 and S4).

### Development of a stem-immune risk score associated with neoadjuvant therapy

To investigate the combined effects of tumor stemness and immune characteristics in patients with GC undergoing neoadjuvant therapy, we collected transcriptome sequencing data from 141 postoperative specimens from patients with GC treated with neoadjuvant therapy at our center. Following neoadjuvant therapy, the tumor regression grade (TRG) of these patients was assessed by pathological evaluation (Fig. S5B). TRG 0 and TRG 1 stages were defined as responders to neoadjuvant chemotherapy, whereas TRG 2 and TRG 3 stages were classified as non-responders. Subsequently, we identified overlapping differentially expressed genes between stemness and immune characteristics and performed Weighted Gene Co-Expression Network Analysis on these genes, representing the shared features of both traits (Figs. 1G, H and S6A–C). The clinical information of the included neoadjuvant therapy samples included responses to neoadjuvant chemotherapy; T, N, and M staging; and AJCC stage (Fig. 1H). Seven gene modules were identified and their relevant characteristics were visualized (Fig. 1H). Notably, eigengenes in the brown module (MEbrown) were significantly associated with neoadjuvant chemotherapy (NAC) benefits (Fig. 1H). Further functional enrichment analysis revealed significant enrichment of pathways such as PD-L1 expression, PD-1 checkpoint pathway in cancer, Wnt signaling pathway, and T cell receptor signaling pathway in the Brown Module. This suggests that these pathways are likely involved in the critical interaction between stemness and immunity in NAC resistance (Fig. S6D).

**Fig. 2:**
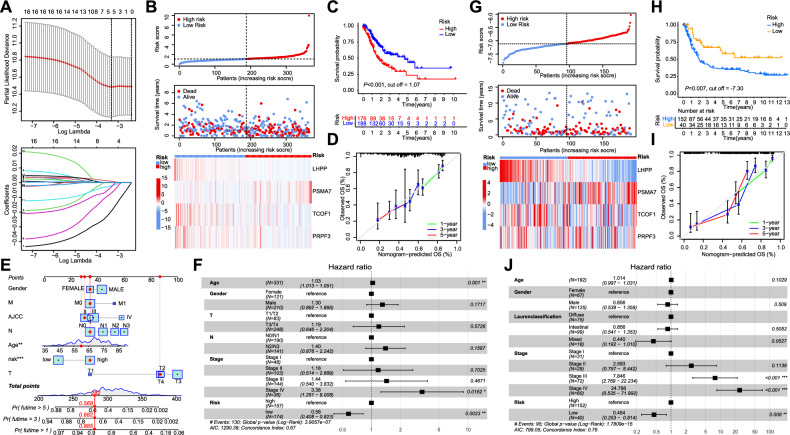
Establishment of the stem-immune risk score and its prognostic value in gastric cancer patients. **A** The optimal lambda and corresponding coefficients for the four indicators were determined using Least Absolute Shrinkage and Selection Operator (LASSO) regression. **B** The stepwise multivariable Cox proportional hazards regression model was applied to generate a risk score for each gastric cancer patient in the TCGA cohort, with patients classified based on the median risk score. **C** Kaplan–Meier survival analysis illustrates the relationship between the risk score and clinical prognosis of gastric cancer patients in the TCGA cohort. The grouping of risk score is based on the optimal cutoff value of 1.07. **D** Time-dependent calibration curves confirmed the accuracy of the risk model in predicting the prognosis of gastric cancer patients in the TCGA cohort. **E** The nomogram-based Cox regression model was constructed to develop a composite score incorporating the risk score, gender, age, and TNM stage for patients in the TCGA cohort. **F** The forest plot displays the hazard ratios from univariate Cox regression analyses for the risk score, gender, age, and TNM stage in the TCGA cohort. **G** The stepwise multivariable Cox proportional hazards regression model was applied to generate a risk score for each gastric cancer patient in the GSE15459 cohort, with patients classified based on the median risk score. **H** Kaplan–Meier survival analysis illustrates the relationship between the risk score and clinical prognosis of gastric cancer patients in the GSE15459 cohort. The grouping of risk score is based on the optimal cutoff value of −7.30. **I** Time-dependent calibration curves confirmed the accuracy of the risk model in predicting the prognosis of gastric cancer patients in the GSE15459 cohort. **J** The forest plot displays the hazard ratios from univariate Cox regression analyses for the risk score, gender, age, and TNM stage in the GSE15459 cohort. *p < 0.05; **p < 0.01; ***p < 0.001.

We selected key genes from the Brown Module and constructed a stem cell-immune risk score using the Least Absolute Shrinkage and Selection Operator algorithm (Fig. 2A). The optimal model included four genes that were significantly associated with risk score (*LHPP*, *PSMA7*, *TCOF1*, and *PRPF3*). Based on this model, patients with TCGA GC were stratified into high- and low-risk groups, with the high-risk group showing a significant association with poorer overall survival (*p* < 0.001, based on the optimal cutoff value of 1.07). Figure 2D shows the robust predictive power of the risk score for the prognosis of patients with GC in TCGA cohort. A nomogram incorporating seven readily available clinical features was developed to guide individualized management of patients with GC (Fig. 2E). In univariate Cox regression analysis, the stem immune risk score was identified as an independent prognostic factor for patients with GC (Fig. 2F). Additionally, we incorporated transcriptome sequencing data from the GSE15459 GC cohort for validation, where we similarly confirmed the prognostic value of the stem cell immune risk score (Fig. 2G–J). Tables S1 and S2 present the relationship between risk scores and baseline characteristics in the two cohorts.

In summary, we identified four key molecules related to the combination of tumor stemness and immune characteristics in neoadjuvant-treated GC, and demonstrated the prognostic value of the risk model they constitute in predicting outcomes for patients with GC post-surgery. Furthermore, we explored the predictive role of this risk model in determining the benefits of neoadjuvant therapy and its involvement in stemness, immune landscapes, and multi-omics events.

### Stemness, immune landscape and multi-omics events of stem-immune risk score

**Fig. 3:**
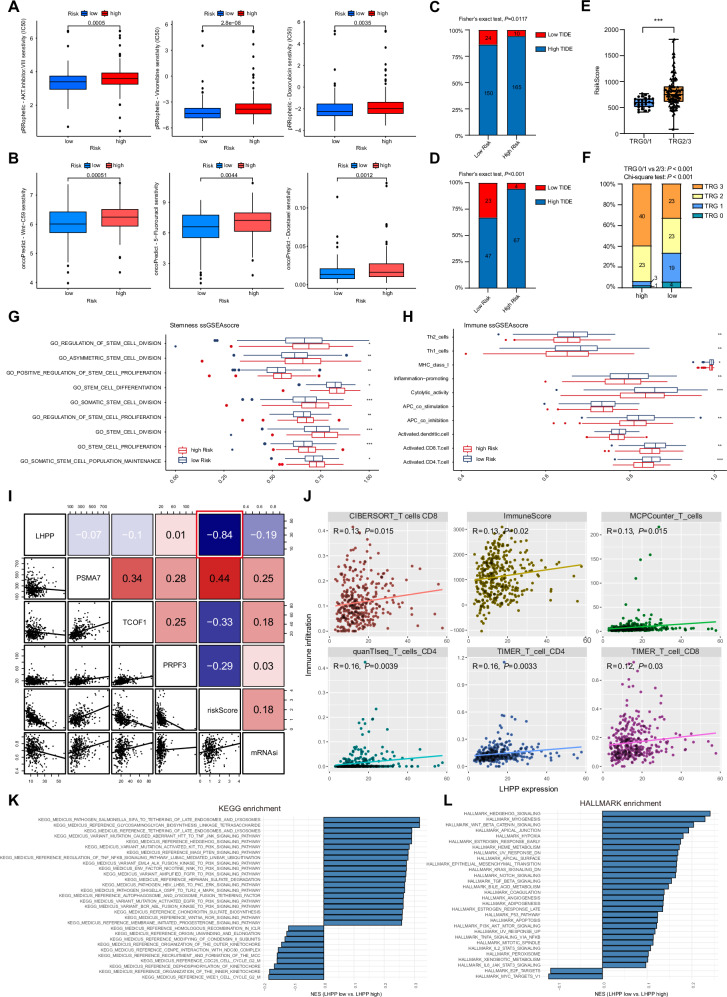
Comprehensive correlation analysis of the risk score with drug resistance, immunity, and stemness. **A**, **B** Using the R packages ‘pRRophetic’ and ‘oncoPredict,’ we estimated the IC50 values for commonly used chemotherapeutic drugs in gastric cancer patients from TCGA database. The upper and lower edges of the box represent the interquartile range, the line within the box indicates the median, and the dots denote outliers. Student’s *t* test was used to compare the statistical difference between the two groups. **C** Fisher’s exact test was used to compare the proportions of TIDE scores between high and low risk groups in the TCGA cohort. **D** Fisher’s exact test was used to compare the proportions of TIDE scores between high and low risk groups in the neoadjuvant therapy cohort from our center. **E** A Student’s *t* test was conducted to compare the differences in risk scores between TRG 0/1 and TRG 2/3 groups in the neoadjuvant therapy cohort from our center. **F** The proportions of TRG grades across different risk groups were compared within the neoadjuvant therapy cohort from our center. **G**, **H** The ssGSEA algorithm was used to compare stemness pathway enrichment scores and immune cell infiltration scores between the high and low risk groups. Student’s *t* test was used to compare the statistical difference between the two groups. **I** Scatter plots illustrate the correlations among *LHPP*, *PSMA7*, *TOCF1*, *PRPF3*, the risk score, and mRNAsi. All correlation analyses were performed using Pearson’s correlation coefficient for statistical analysis. **J** Scatter plots depict the correlation between *LHPP* expression and immune cell infiltration levels across various algorithms. All correlation analyses were performed using Pearson’s correlation coefficient for statistical analysis. **K**, **L** GSVA enrichment analysis demonstrated the pathway enrichment profiles of KEGG and HALLMARK pathways across different levels of *LHPP* expression. *p < 0.05; **p < 0.01; ***p < 0.001.

Using the pRRophetic and oncoPredict drug resistance prediction algorithms, we found that patients with a low stem cell-immune risk score exhibited a significantly better response to various clinical chemotherapeutic agents, as well as increased drug sensitivity to AKT inhibitors and Wnt-C59 (Fig. 3A, B). These results suggested that the stem cell-immune risk score may reflect the pivotal role of the Akt/Wnt/β-catenin signaling pathway in mediating chemotherapy resistance in GC, similar to the enrichment of the ‘Wnt signaling pathway’ observed in Fig. S6D. Given the previously demonstrated prognostic value of the stem cell-immune risk score in predicting postoperative outcomes in patients with GC who did not receive neoadjuvant chemotherapy, we aimed to explore the predictive potential of this risk model in assessing the benefit of neoadjuvant therapy. The tumor immune dysfunction and exclusion (TIDE) algorithm was used to evaluate the likelihood of tumor immune escape based on the gene expression profiles of the tumor samples. We selected patients with GC from the TCGA cohort who had received adjuvant chemotherapy and calculated their TIDE scores. A higher TIDE score for each patient indicated a greater likelihood of tumor immune escape (Fig. S7A, B). The analysis revealed a significantly higher proportion of patients with low TIDE scores in the low-risk group, whereas the high-risk group contained a greater number of patients with elevated TIDE scores (Fisher’s exact test, *p* = 0.0117; Figs. 3C and S7A). Transcriptome sequencing data from 141 patients with GC who received NAC at our center validated these findings, showing that the low-risk group was associated with a lower likelihood of tumor immune escape (Fisher’s exact test, *p* < 0.001; Figs. 3D and S7B). Furthermore, we performed a stratified analysis of patients from our center database based on TRG grading and risk scores. The analysis revealed that patients in the TRG 0/1 group had significantly lower risk scores (*p* < 0.001, Fig. 3E), while patients with GC in the low-risk group demonstrated a significantly greater benefit from NAC (TRG 0/1 vs. 2/3: *p* < 0.001; chi-square test: *p* < 0.001, Fig. 3F). In the ssGSEA of stemness and immunity, the high-risk group was associated with a greater enrichment of stemness pathways and a lower enrichment of anti-tumor immune response cells (Fig. 3G, H). In both the TCGA and GSE15459 cohorts, HALLMARK and Kyoto Encyclopedia of Genes and Genomes (KEGG) enrichment analyses consistently demonstrated that a higher risk score was associated with the enrichment of pathways such as oxidative phosphorylation, AKT signaling, WNT signaling, and metabolic processes (Fig. S7C, D). These findings suggest that the Akt/Wnt/β-catenin and metabolism-related pathways may serve as key mediators of stemness-immune responses contributing to NAC resistance.

To further construct the multi-omics landscape of the risk score, we utilized the ‘maftools’ R package to compare the distribution and frequency of somatic copy number alterations between the high- and low-risk groups. The analysis revealed a significantly higher mutational burden of ARID1A in the high-risk group than that in the low-risk group (Fig. S8A). ARID1A has previously been reported to be associated with enhanced cancer stemness characteristics and tumor immune evasion when mutated. No significant differences were observed between the high- and low-risk groups in terms of variant classification or type (Fig. S8B, C). In the analysis of copy number variations (Fig. S9A–D), we observed a significantly higher frequency of copy number loss for LHPP, one of the key genes in the high-risk group, located at 10q26.3 (Fig. S9D). Given that *LHPP* showed the strongest correlation with the risk score (−0.84, Fig. 3I), it is highly likely that *LHPP* (phospholysine phosphohistidine inorganic pyrophosphate phosphatase) is a key gene mediating the response to neoadjuvant chemotherapy. Given the pivotal role of *LHPP* in the risk score, further research on its function within the tumor immune microenvironment and cancer cell stemness characteristics is of critical importance.

### LHPP regulates anti-tumor immune cell infiltration within the tumor microenvironment

Using the CIBERSORT, ESTIMATE, MCPCounter, quanTIseq, and TIMER algorithms, we calculated the immune cell infiltration levels in TCGA patients with GC. The analysis revealed a significant positive correlation between *LHPP* mRNA expression and the infiltration of various antitumor immune cells (Figs. 3J and S10A). KEGG and HALLMARK enrichment analyses of neoadjuvant sequencing data from our center indicated that low *LHPP* expression was associated with the enrichment of anti-tumor immune response pathways, including the PI3K-AKT-mTOR, JAK-STAT, and WNT-β-catenin signaling pathways (Fig. 3K, L). In both TCGA and GSE15459 cohorts, GO and KEGG enrichment analyses revealed the importance of *LHPP* in tumors through the PI3K-Akt signaling pathway. *LHPP* was also enriched in biological processes related to extracellular matrix remodeling, immune cell activation, and chemotaxis (Fig. S10B–E).

**Fig. 4:**
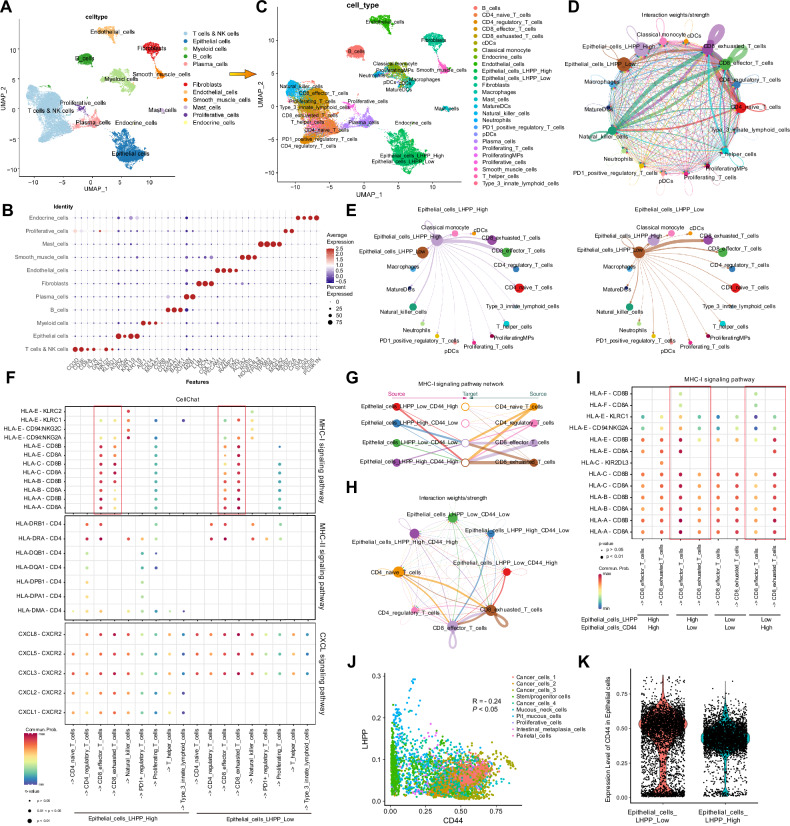
Analysis of LHPP in relation to immunity and stemness in the single-cell data from our center’s neoadjuvant therapy cohort. **A** The UMAP plot illustrates the major cell types identified in the single-cell transcriptomic dataset from the neoadjuvant therapy cohort at our centre, including T & NK cells, epithelial cells, myeloid cells, B cells, plasma cells, fibroblasts, endothelial cells, smooth muscle cells, proliferative cells, mast cells, and endocrine cells. **B** Dot plot showing the average expression and percentage of specific markers across different cell types, highlighting cell-type-specific marker genes. The color intensity represents average expression levels, and the dot size indicates the percentage of cells expressing each marker. **C** The UMAP plot displays the subpopulations identified in the single-cell transcriptomic dataset from the neoadjuvant therapy cohort at our centre, including B cells, CD4+ naive T cells, CD4+ regulatory T cells, CD8+ effector T cells, CD8+ exhausted T cells, conventional dendritic cells (cDCs), classical monocytes, endocrine cells, endothelial cells, epithelial cells (*LHPP* High and *LHPP* Low), fibroblasts, macrophages, mast cells, mature dendritic cells (MatureDCs), natural killer cells, neutrophils, PD1+ regulatory T cells, plasmacytoid dendritic cells (pDCs), plasma cells, proliferating T cells, proliferating myeloid progenitors (ProliferatingMPs), proliferative cells, smooth muscle cells, T helper cells, and type 3 innate lymphoid cells. **D** Network plot displaying the interaction weights and strengths between epithelial cell subpopulations and various immune cells, as analyzed by CellChat. **E** A simplified network diagram focusing on the signaling interactions between *LHPP*^high^ and *LHPP*^low^ epithelial cell subpopulations and immune cells, illustrating distinct communication patterns. **F** Dot plot showing the specific interactions between epithelial cell subgroups and immune cells through the MHC-I, MHC-II, and CXCL signaling pathways. The size of each dot represents the communication probability, and color intensity indicates the *p* value significance level. **G**, **H** Network plot depicting the MHC-I signaling pathway interactions among epithelial cell subgroups and various CD4+ and CD8+ T cell subtypes. The thickness of the connections represents the interaction strength, highlighting the elevated communication strength between *LHPP*^low^ *CD44*^high^ epithelial cells and CD8+ exhausted T cells, as well as between *LHPP*^high^ *CD44*^low^ epithelial cells and CD8+ effector T cells. **I** Dot plot comparing MHC-I pathway-mediated communication with CD8+ T cell subtypes across different *LHPP* and *CD44* expression subgroups in epithelial cells. Significant variations in communication patterns are evident, particularly highlighting the interactions involving HLA molecules (HLA-A, HLA-B, HLA-C) and various CD8+ T cell markers. **J** Scatter plot displaying the correlation between *LHPP* and *CD44* expression across various epithelial cell types, including cancer cells, stem/progenitor cells, and mucus neck cells. **K** Violin plot illustrating the differential expression of *CD44* in epithelial cells based on *LHPP* expression levels. *p < 0.05; **p < 0.01; ***p < 0.001.

Additionally, we performed single-cell sequencing of postoperative specimens from ten patients in our center who received neoadjuvant therapy at our center. These included five patients who responded to the therapy (two with TRG 0 and three with TRG 1) and five patients who were resistant to the therapy (three with TRG 3 and two with TRG 2) (Fig. 4A). Based on the marker genes of each cluster, the cells were annotated into the following groups: T and NK cells, epithelial cells, myeloid cells, B cells, plasma cells, fibroblasts, endothelial cells, smooth muscle cells, mast cells, proliferative cells, and endocrine cells (Fig. 4A, B). We further subdivided T and NK cells, myeloid cells, and epithelial cells based on their respective cluster markers (Figs. S11A–F and 4C). In the annotation and clustering of GC epithelial cells, we found that *LHPP* was widely expressed across various GC epithelial cell subpopulations, with the highest expression observed in pit mucous cells (Fig. S12A, B). In the annotation and clustering of GC epithelial cells, *LHPP* was broadly expressed across various epithelial cell subpopulations, with the highest expression observed in pit mucous cells. To study the communication between *LHPP* in epithelial cells and immune cells in the tumor microenvironment, epithelial cells were divided into two groups based on the median expression level of *LHPP*: *LHPP*^high^ epithelial cells and *LHPP*^low^ epithelial cells (Fig. S12C–E). T cell, myeloid, and epithelial cell subpopulations were included, and CellChat was used to analyze intercellular communication (Fig. 4D). Cellular communication analysis revealed stronger interactions between *LHPP*^high^ epithelial cells and CD8+ effector T cells, whereas *LHPP*^low^ epithelial cells showed stronger interactions with CD8+ exhausted T cells. This suggests that *LHPP*^high^ epithelial cells play a critical role in the regulation of antitumor immune responses. Furthermore, we visualized the communication networks of MHC class I and II signals between epithelial cells with different *LHPP* expression levels and immune cells. The analysis indicated that *LHPP*^low^ epithelial cells primarily communicated with CD8+ exhausted T cells via the MHC class I signaling pathway, whereas *LHPP*^high^ epithelial cells predominantly interacted with CD8+ effector T cells via the same pathway (Fig. S13A). There were no significant differences in cellular communication between the two groups via the MHC class II signaling pathway (Fig. S13B). The bubble plot illustrates the differences in signaling communication between epithelial cells with varying *LHPP* expression levels, CD8+ effector T cells, and CD8+ exhausted T cells (Fig. 4F). As single-cell samples included data on NAC responses, we performed a comparative analysis of cellular communication between samples that responded and those that did not. Enhanced cellular communication signals in the responder group are represented by blue lines, whereas those in the non-responder group are indicated by red lines (Fig. S13C, D). Figure S13D shows that, in the NAC-responsive group, communication between *LHPP*^high^ epithelial cells and CD8+ effector T cells was significantly enhanced (blue lines), whereas communication between *LHPP*^low^ epithelial cells and CD8+ exhausted T cells was notably increased (red lines). Further visualization of MHC class I signaling pathways revealed differences in cellular communication between the responder and non-responder groups. In the non-responder group, *LHPP*^low^ epithelial cells exhibited strong signaling communication with CD8+ exhausted T cells via the MHC class I pathway, whereas in the responder group, *LHPP*^high^ epithelial cells showed stronger communication with CD8+ effector T cells through the same pathway (Fig. S13E, F).

These findings suggest that high *LHPP* expression in GC regulates CD8+ effector T cell infiltration and function, thereby modulating the immune landscape of the tumor microenvironment and mediating the sensitivity of GC cells to neoadjuvant chemotherapy.

### Loss of LHPP in gastric adenocarcinoma drives stem cell-like characteristics via GSK3β phosphorylation

Epithelial cells were classified into *CD44*^high^ and *CD44*^low^ based on the median expression level of *CD44* (Fig. S12G, I). The expression distribution of *LHPP* and *CD44* in epithelial cells exhibited a mutually exclusive relationship (Fig. S12F, J) with a clear negative correlation between their expression levels (Fig. 4J). In epithelial cells with low *LHPP* expression, *CD44* expression was significantly elevated (Fig. 4K). Epithelial cells were grouped based on *LHPP* and *CD44* expression levels, resulting in four subgroups: *LHPP*^low^ *CD44*^high^ epithelial cells, *LHPP*^high^ *CD44*^low^ epithelial cells, *LHPP*^low^ *CD44*^low^ epithelial cells, and *LHPP*^high^ *CD44*^high^ epithelial cells (Fig. 4G). Analysis of the cellular communication between these four epithelial subgroups and CD4+ and CD8+ T cells revealed that *LHPP*^low^ *CD44*^high^ epithelial cells exhibited the strongest communication with CD8+ exhausted T cells, whereas *LHPP*^high^ *CD44*^low^ epithelial cells showed the strongest communication with CD8+ effector T cells (Fig. 4G, H). Differences in cellular communication with CD8 + T cells were observed among the four groups in the MHC class I signaling pathway. *LHPP*^high^ *CD44*^low^ epithelial cells exhibited greater communication with CD8+ effector T cells through the MHC class I pathway than *LHPP*^low^ *CD44*^high^ epithelial cells did (Fig. 4I).

**Fig. 5:**
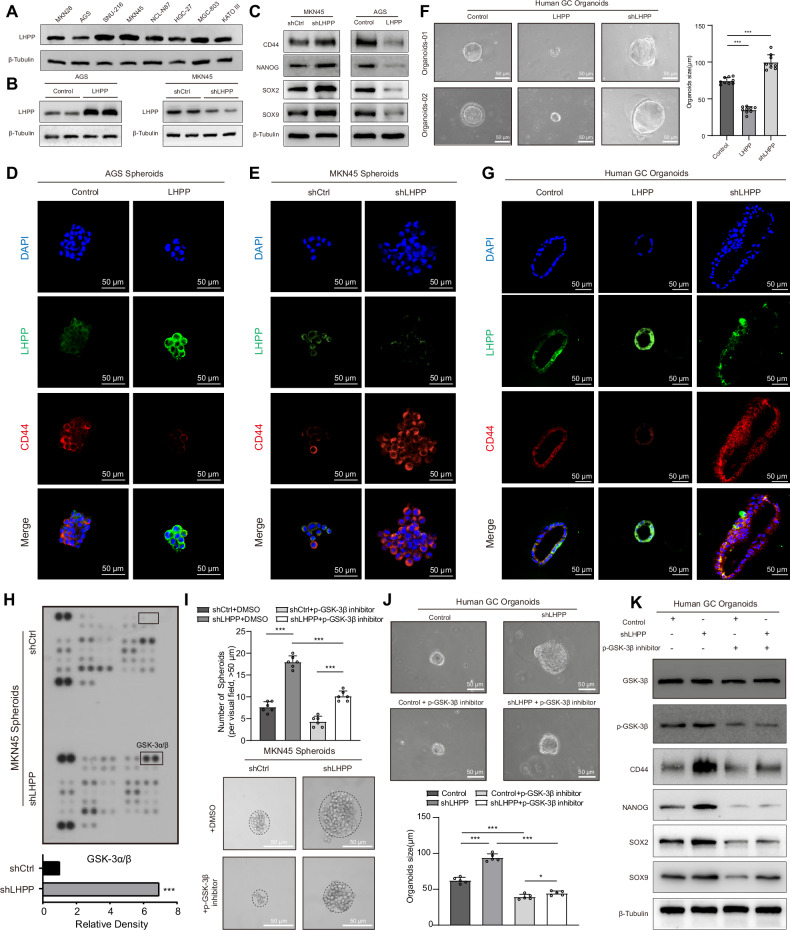
LHPP inhibited stem cell-like characteristics in gastric cancer cells in vitro. **A** Basic protein expression of *LHPP* in gastric cancer cell lines was detected by western blotting. **B** Stable *LHPP*-overexpressing AGS cells and *LHPP*-knockdown MKN45 cells were constructed. Western blotting confirmed the changes in *LHPP* expression. **C** Western blot analysis was used to detect the protein expression levels of *CD44*, *NANOG*, *SOX2*, and *SOX9* in gastric cancer cells with *LHPP* knockdown or overexpression. **D** Immunofluorescence imaging shows the expression of *LHPP* and *CD44* in AGS cells overexpressing *LHPP*. **E** Immunofluorescence imaging shows the expression of *LHPP* and *CD44* in *LHPP*-knockdown MKN45 cells. **F** The impact of *LHPP* overexpression or knockdown on the growth of patient-derived gastric cancer organoids. Organoid diameters were quantitatively analyzed, and a Student’s *t* test was used to compare the differences between the two groups. **G** Immunofluorescence imaging shows the expression of *LHPP* and *CD44* in patient-derived gastric cancer organoids with *LHPP* overexpression or knockdown. **H** Human phospho-kinase microarray analysis of conditioned medium from MKN45 cells with stable *LHPP* knockdown. The relative signal intensities of the indicated proteins are summarized below. **I** Spheroid formation was assessed in MKN45 cells transfected with sh*LHPP* and treated with the GSK-3β inhibitor Laduviglusib (CHIR-99021; 10 µM). Student’s *t* test was used to compare the differences between the two groups. **J** Organoid size was assessed in patient-derived organoids transfected with *LHPP* knockdown and treated with the GSK-3β inhibitor Laduviglusib (CHIR-99021; 10 µM). Student’s *t* test was used to compare the differences between the two groups. **K** Western blot analysis was used to detect the protein expression levels of *CD44*, *NANOG*, *SOX2*, and *SOX9* in patient-derived organoids transfected with *LHPP* knockdown or treated with the GSK-3β inhibitor. *p < 0.05; **p < 0.01; ***p < 0.001.

**Fig. 6:**
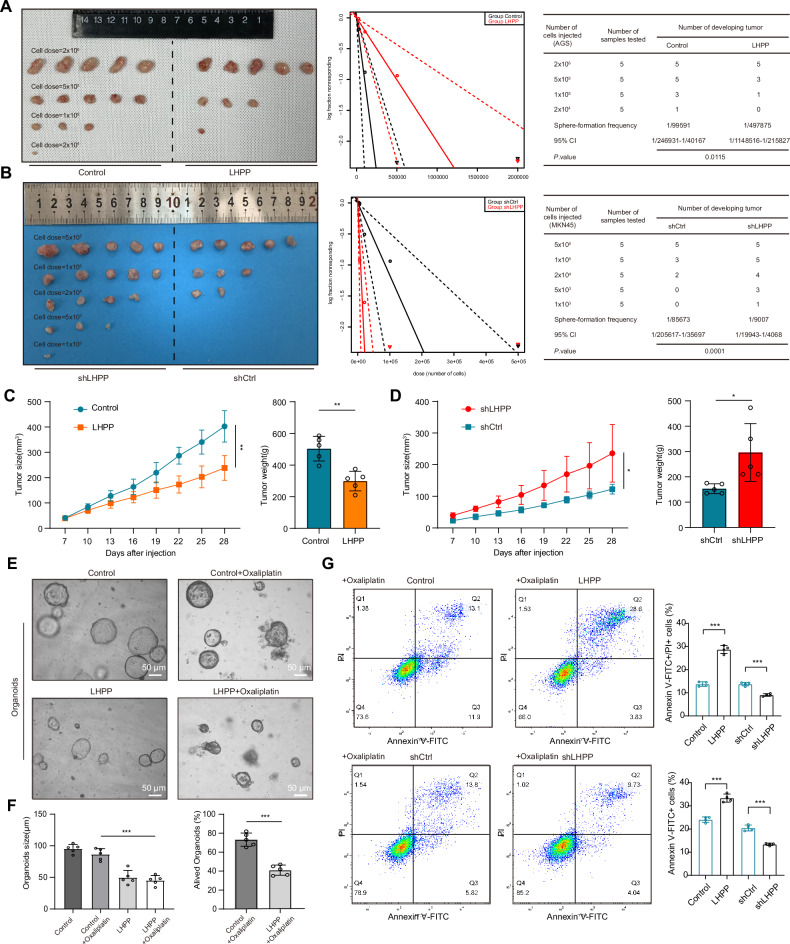
LHPP downregulation enhances stem cell-like characteristics and chemoresistance in gastric cancer cells. **A** Tumor formation frequency at varying cell inoculation densities in xenograft models of AGS cells with upregulated *LHPP* expression. **B** Tumor formation frequency at different cell inoculation densities in xenograft models of MKN45 cells with downregulated *LHPP* expression. **C** Tumor weight and volume changes were recorded in xenograft models of AGS cells with a cell dose of 2 × 10^6^. Student’s *t* test was used to compare the differences between the two groups. **D** Tumor weight and volume changes were recorded in xenograft models of MKN45 cells with a cell dose of 5 × 10^5^. Student’s *t* test was used to compare the differences between the two groups. **E** Survival of patient-derived gastric cancer organoids with stable *LHPP* overexpression, with and without oxaliplatin (OXA) treatment. **F** Quantitative analysis of the number and size of surviving gastric cancer organoids. Data are presented as mean ± standard deviation (mean ± SD), with analysis performed using a Student’s *t* test. **G** Apoptosis in *LHPP*-overexpressing and *LHPP*-knockdown gastric cancer cells following OXA treatment was assessed using apoptosis flow cytometry. Bar charts provide a quantitative analysis of the differences in Annexin V-FITC and PI double-positive cell percentages. Student’s *t* test was used to compare the differences between the two groups. *p < 0.05; **p < 0.01; ***p < 0.001.

In summary, epithelial cell subgroups with different LHPP and CD44 expression levels exhibited significant differences in communication with CD8^+^ T cells. *LHPP* and *CD44* expression levels significantly influence the communication patterns between epithelial and immune cells. We performed further experiments to validate the role of *LHPP* in promoting the stem cell-like characteristics of GC cells. Based on the expression levels of *LHPP* in different GC cell lines, we established *LHPP*-overexpressing AGS GC cells and *LHPP*-knockdown MKN45 GC cells (Fig. 5A, B). Low *LHPP* expression led to a significant increase in the protein levels of stemness markers, such as *CD44*, *NANOG*, *SOX2*, and *SOX9* in GC cell lines, whereas high *LHPP* expression suppressed the expression of these stemness markers (Fig. 5C). The 3D spheroid formation assay demonstrated that *LHPP* overexpression reduced the spheroid-forming ability of AGS cells (Fig. 5D) and significantly decreased *CD44* expression (Fig. S14A). Conversely, *LHPP* knockdown enhanced the spheroid-forming capacity of MKN45 cells (Fig. 5E), which was accompanied by a significant increase in *CD44* expression (Fig. S14A). Flow cytometry analysis showed that high *LHPP* expression significantly reduced the proportion of *CD44*-positive GC cells, whereas low *LHPP* expression markedly increased the proportion of *CD44*-positive cells (Fig. S14B, C). Limiting dilution assays revealed that the spheroid-forming capacity of *LHPP*-knockdown cells increased from 1/4.02–1/1.97 to 1/1.98–1/1.22 (AGS cells, *p* = 0.0012, Fig. S14D). In contrast, the spheroid-forming ability of *LHPP*-overexpressing cells decreased from 1/3.41–1/1.69 to 1/5.26–1/2.62 (MKN45 cells, *p* = 0.0279, Fig. S14E). Organoid experiments derived from patients with GC demonstrated that the diameter of organoids transfected with the *LHPP*-overexpressing virus was significantly reduced, whereas those transfected with the *LHPP*-knockdown virus exhibited a marked increase in diameter (Fig. 5F). Fluorescence staining of organoids revealed that high *LHPP* expression significantly inhibited organoid size and the proportion of CD44-positive cells, whereas low *LHPP* expression had the opposite effect (Figs. 5G and S14F). To further elucidate the pathways through which *LHPP* influences stem cell-like characteristics, we used a human phosphokinase array kit to identify the phosphorylation kinases that are closely associated with *LHPP* (Fig. 5H). The results indicated differential expression levels of several phosphorylated protein kinases between control cells and cells with low *LHPP* expression, with significant changes in phosphorylated GSK-3β (Fig. 5H). We hypothesized that low *LHPP* expression in GC cells mediates stem cell-like characteristics via GSK-3β phosphorylation. The addition of a phosphorylated GSK-3β inhibitor significantly reversed the enhanced spheroid formation ability in *LHPP* low-expression cells and stem cell-like characteristics observed in human organoids (Fig. 5I, J). Additionally, western blotting confirmed that the phosphorylated GSK-3β inhibitor restored the elevated expression of *CD44*, *NANOG*, *SOX2*, and *SOX9* in human organoids with low *LHPP* expression (Fig. 5K). Conversely, a phosphorylated GSK-3β activator reversed the reduced spheroid formation ability observed in *LHPP* high-expressing cells (Fig. S14G). Subcutaneous injection of *LHPP*-overexpressing, *LHPP*-knockdown, and the corresponding control cell lines was performed in nude mice, and tumor formation rates and numbers were recorded. Compared to the control group, *LHPP*-overexpressing AGS GC cells showed a statistically significant reduction in the tumor formation rate and number (Fig. 6A), as well as a decrease in tumor weight and volume (Fig. 6C). Conversely, *LHPP*-knockdown MKN45 GC cells exhibited a statistically significant increase in tumor formation rate and number (Fig. 6B), along with increased tumor weight and volume, compared to the control group (Fig. 6D). To verify whether *Lhpp* similarly regulates tumor growth and stemness characteristics in wild-type background mice, we collected four mouse GC cell lines: YTN2, YTN3, YTN5, and YTN16. Based on the expression levels of *Lhpp* in the four mouse GC cell lines, we constructed *Lhpp*-overexpressing YTN5 mouse GC cells and *Lhpp*-knockdown YTN3 mouse GC cells (Fig. S15A). The tumor formation rate and number of YTN5 GC cells overexpressing *Lhpp* in C57BL/6 mice were significantly lower than those in the control group, with reduced tumor weight and volume (Fig. S15B, D). In contrast, the tumor formation rate, quantity, tumor weight, and volume of *Lhpp*-knockdown YTN3 GC cells were increased (Fig. S15C, E). These findings suggest that *LHPP* significantly inhibits robust tumor growth and stem cell-like characteristics in vivo. To investigate the effect of *LHPP* on chemoresistance of GC cells, we added oxaliplatin (OXA) to human GC organoids (Fig. 6E). Following OXA treatment, organoids overexpressing *LHPP* exhibited a significant reduction in size and survival rate compared to the control group (Fig. 6F). OXA was then added to the GC cell lines with either *LHPP* overexpression or knockdown at 50% confluence. Flow cytometry analysis showed that *LHPP* overexpression significantly increased the apoptotic rate of GC cells under OXA treatment, whereas *LHPP* knockdown had the opposite effect (Fig. 6G).

### Validation of LHPP expression in mouse tumor models and clinical samples

**Fig. 7:**
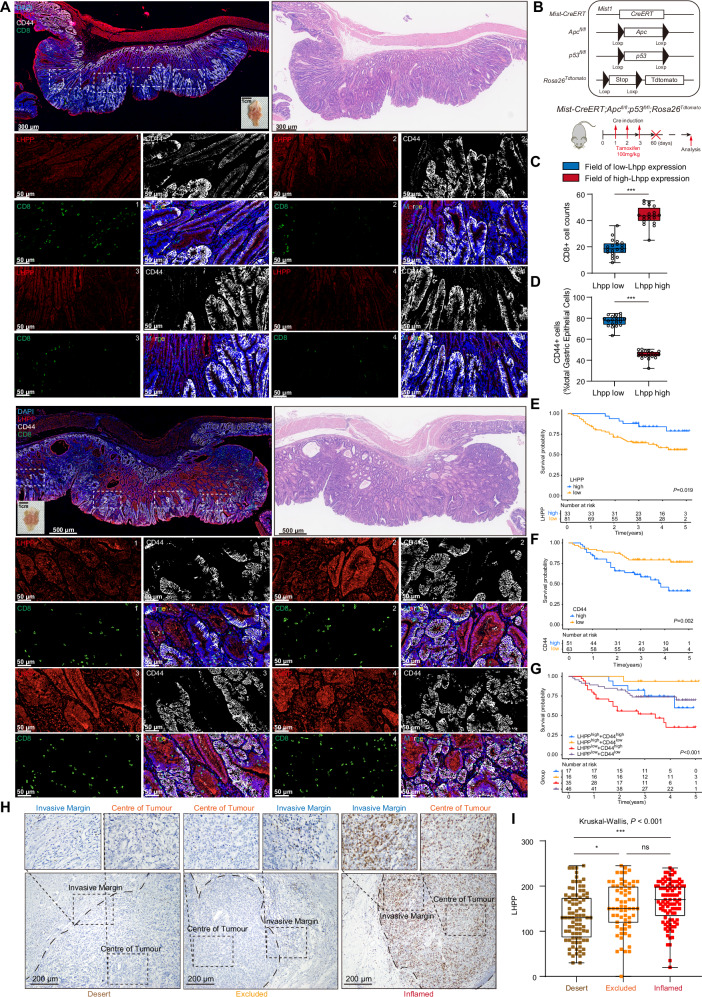
Expression of LHPP in tumors is associated with stemness characteristics, the anti-tumor immune microenvironment, and prognosis. **A** Representative images of gastric cancer in the *Mist-CreERT;Apc*^*fl/fl*^*;p53*^*fl/fl*^*;Rosa26*^*Tdtomato*^ conditional knockout gene mouse model. Multiplex immunofluorescence staining was used to assess the expression of *LHPP*, *CD44*, CD8, and DAPI in the model. The upper right corner displays HE-stained images of the gastric cancer region in the mouse model. **B** The experimental strategy for genetic recombination in the *Mist-CreERT;Apc*^*fl/fl*^*;p53*^*fl/fl*^*;Rosa26*^*Tdtomato*^ mouse model. **C** Statistical analysis of CD8+ T cell counts in fields of *Lhpp* low-expression and *Lhpp* high-expression regions. A total of 20 regions from four mouse gastric cancer models were analyzed (five randomly selected regions per mouse), including 10 *Lhpp* low-expression regions and 10 *Lhpp* high-expression regions. The distinction between *Lhpp* high and low expression was determined based on the median H-score of *Lhpp*. Statistical differences between the two groups were compared using Student’s *t* test. **D** Statistical analysis of the number of CD44+ gastric epithelial cells in fields of *Lhpp* low-expression and *Lhpp* high-expression regions. **E** Overall survival curves for gastric cancer patients with low and high *LHPP* expression. **F** Overall survival curves for gastric cancer patients with low and high *CD44* expression. **G** Kaplan–Meier survival curves depicting overall survival probabilities for gastric cancer patients based on combined *LHPP* and *CD44* expression levels. Patients are classified into four groups: *LHPP*^high^ *CD44*^high^, *LHPP*^high^ *CD44*^low^, *LHPP*^low^ *CD44*^high^, and *LHPP*^low^ *CD44*^low^. **H** Representative IHC images showing CD8 expression in the tumor center and invasive margin across three immune phenotypes: Desert, Excluded, and Inflamed. Magnified areas highlight differences in CD8 staining among the phenotypes. Scale bar = 200 μm. **I** Box plot illustrating *LHPP* expression levels across Desert, Excluded, and Inflamed immune phenotypes. Student’s *t* test was used to compare the statistical difference between the two groups. Statistical significance was determined using the Kruskal–Wallis test. *p < 0.05; **p < 0.01; ***p < 0.001.

We used the *Mist-CreERT;Apc*^*fl/fl*^*;p53*^*fl/fl*^*;Rosa26*^*Tdtomato*^ conditional knockout gene mouse model (Figs. 7A, and S16A, B), with the corresponding genetic mouse induction model shown in Fig. 7B. Mouse tumors are presented with HE staining to ensure they are all mouse cancer samples. As shown in Fig. 7A, we used this model to compare the expression and distribution of *Lhpp*, *Cd44*, and CD8 in GC. We observed that *Cd44* expression was significantly increased in areas with low *Lhpp* expression, and there was an exclusion between the localization of *Cd44* and *Lhpp* (Figs. 7A and S16A, B). Furthermore, areas with low *Lhpp* expression were accompanied by a reduction in the infiltration of CD8 + T cells within the tumor. In contrast, with high *Lhpp* expression, *Cd44* expression significantly decreased, and CD8 + T cell infiltration into the tumor was significantly enhanced (Figs. 7A and S16A, B). We further supported the negative correlation between *Lhpp* and *Cd44*, as well as the positive correlation between *Lhpp* and CD8 infiltration, through statistical analysis of multiple regions across several mice (Fig. 7C, D).

Immunohistochemistry (IHC) was performed to assess the expression of *LHPP* and *CD44* in postoperative specimens from patients with GC (Fig. S17A). In patients with GC, high *LHPP* expression was significantly associated with a favorable prognosis, whereas high *CD44* expression was correlated with a poorer prognosis (Figs. 7E, F and S18B, C). *LHPP* expression was significantly and negatively correlated with *CD44* expression (Fig. S17A, B). Combined analysis of *LHPP* and *CD44* expression revealed that high *LHPP* expression was significantly associated with better survival prognosis, particularly in the context of low *CD44* expression. Conversely, low *LHPP* and high *CD44* expression were significantly correlated with poor survival prognosis (*p* < 0.001). These findings suggest that the expression status of *LHPP* and *CD44* may serve as independent or combined biomarkers for predicting patient survival (Figs. 7G and S18D). Furthermore, we investigated changes in immune cell composition within the tumor microenvironment mediated by *LHPP* in clinical samples. We classified the patients into three immune groups: ‘inflamed,’ ‘excluded,’ and ‘desert’. IHC was used to assess CD8+ immune cell expression in the tumor center and invasive margins across these groups, thereby validating immune cell infiltration patterns (Fig. 7H). The analysis revealed significant differences in *LHPP* expression among the three immune groups, with notably higher *LHPP* expression in the inflamed group (Fig. 7I). We quantitatively assessed the infiltration of various tumor immune cells in the tumor center and invasive margins, including CD45+ leukocytes, CD3+ and CD8+ cytotoxic T cells, CD4+ helper T cells, CD45RO+ activated and memory T cells, and FOXP3+ regulatory T cells (Fig. S17C–T). Overall, high *LHPP* expression was significantly associated with altered tumor immune infiltration characteristics, showing a positive correlation with the infiltration abundance of CD3+ (Fig. S17C–E), CD4+ (Fig. S17F–H), CD45+ T cells (Fig. S17I–K), and CD8+ (Fig. S17R–T), in both the center of the tumor and the invasive margin.

In summary, high LHPP expression is significantly associated with a favorable prognosis and enhanced antitumor immune microenvironment in patients with GC, particularly marked by increased infiltration of CD8+ cytotoxic T cells and other T cell subsets. These findings suggest that *LHPP* may serve as a biomarker for predicting survival outcomes and immune status in patients with GC.

## Discussion

Tumorigenicity and immune evasion are closely related, and their interaction may lead to the reduced efficacy of immunotherapy. GC cells with high PD-L1 expression may be more inclined to evade the immune system, thereby promoting tumor progression and worsening prognosis [28]. Additionally, the tumor microenvironment plays a significant role in tumorigenicity and immune evasion, with tumorigenic signaling activating immunosuppressive programs that underlie the immune evasion of tumor cells [29]. Consequently, patients with GC in the high tumorigenicity/low immune groups may have a poor prognosis owing to the strong invasiveness, metastatic ability, and immune evasion capabilities of tumor cells. Our study indicated that high *LHPP* expression is closely associated with significant infiltration of antitumor immune cells and suppression of stemness characteristics, suggesting its role as a potential tumor suppressor gene in GC.

Tumorigenicity and immune evasion are key factors in chemoresistance in GC. Our multi-omics analysis further demonstrated that *LHPP* inhibits the stemness characteristics of GC cells and weakens their drug resistance by regulating the phosphorylation pathway of GSK-3β. *LHPP* is a highly conserved histidine phosphatase that functions as a tumor suppressor in various cancers. *LHPP* can inhibit the proliferation, growth, and migration of tumor cells and promote apoptosis by participating in the regulation of various cellular signaling pathways and protein phosphorylation [23, 30]. *LHPP* expression is reduced in GC and its expression level is closely related to patient prognosis. Low *LHPP* expression is associated with poor prognosis in GC, indicating that *LHPP* may serve as a potential prognostic biomarker for patients with GC [21]. Our study showed that *LHPP* plays an important role in inhibiting tumorigenicity and enhancing drug response, a finding that is consistent with that of other studies. Upregulation of *LHPP* may reduce cell stemness by inhibiting the activation of JNK and p38 MAPK signaling pathways, while enhancing the accumulation of intracellular reactive oxygen species, thereby promoting cisplatin-induced apoptosis in GC cells and increasing sensitivity to cisplatin [31]. Additionally, *LHPP* can regulate the chemoresistance of GC cells through specific signaling pathways, such as PI3K-AKT [24] and Wnt-β-catenin [27]. In the PI3K-AKT signaling pathway, downregulation of LHPP expression is associated with chemoresistance in GC cells. Overexpression of *LHPP* can lead to reduced phosphorylation of the PI3K/AKT/mTOR pathway, whereas depletion of *LHPP* has the opposite effect [24]. *LHPP* may enhance the sensitivity of GC cells to chemotherapeutic drugs by inhibiting the phosphorylation pathway of GSK-3β.

The stemness characteristics of tumor cells are closely related to their self-renewal and repopulating abilities, which enable them to resist conventional treatments and make them prone to recurrence. Furthermore, the Wnt/β-catenin signaling pathway may enhance the chemoresistance of GC cells by promoting their stemness characteristics. In colorectal cancer, *LHPP* inhibits the migration and invasion of cancer cells by suppressing the phosphorylation of Smad3 in the TGF-β pathway [26]. Qin et al. showed that upregulating the expression of *LHPP* can activate the immunogenicity of tumor cells and the transition to ferroptosis, thereby achieving effective treatment of hepatocellular carcinoma [32]. These studies support the important role of *LHPP* in the inhibition of tumorigenicity, and provide potential targets for the development of new therapeutic strategies.

This study also revealed for the first time a significant communication relationship between *LHPP* low-expressing epithelial cell subgroups and CD8+ exhausted T cells, suggesting a potential mechanism for immune evasion. An increase in CD8+ T cell infiltration in the tumor microenvironment is associated with better prognosis, and CD8+ T cells have the ability to selectively detect and eliminate cancer cells. Numerous studies have shown that T cell infiltration has an important impact on the efficacy of immunotherapy [33, 34]. Although immunotherapy has made significant progress in some tumors in recent years, a considerable number of patients with GC still do not benefit from immunotherapy, which may be related to tumor immune suppression [35]. Stem cell-like characteristics in the tumor microenvironment create an environment that allows tumor cells to escape host immune surveillance, thereby resisting cancer treatment [36–38]. Our study showed that *LHPP* expression may be related to the infiltration of immune cells into the tumor microenvironment, suggesting that *LHPP* may regulate the immune microenvironment through multiple mechanisms. Zhou et al. found that oxidative phosphorylation of *LHPP* is associated with increased immune infiltration in osteosarcoma [30]. Based on stratified analysis of *LHPP* and *CD44*, we found that gastric epithelial cells with high *LHPP* and low *CD44* levels exhibited strong signal communication with CD8+ effector T cells. This indicates that *LHPP* plays a key role in regulating the infiltration of antitumor immune cells, and that changes in its expression and activity may have a significant impact on the immune evasion of tumors and the effectiveness of immunotherapy.

*CD44* is a transmembrane glycoprotein and its overexpression in tumor stem cells is associated with the occurrence and development of tumors [39]. Dysregulation of *CD44* expression is closely associated with tumor proliferation, invasion, metastasis, and therapeutic resistance [40]. The expression of *CD44* in various solid tumors, such as colorectal cancer [41], lung cancer [42], and Ewing’s sarcoma [43], differs from that in normal tissues, indicating its important role in tumorigenesis. *CD44* is a promising candidate for predicting the prognosis of patients with malignant tumors. Hou et al. [44] found that *CD44* is highly expressed in GC tissues compared with normal tissues and promotes the proliferation and migration of GC cells. In this study, the overall survival, progression-free survival, and post-progression survival were prolonged in the group with low *CD44* expression, indicating that high *CD44* expression in GC is an independent prognostic factor related to immune invasion and is associated with poor prognosis in GC. Gama et al. [45] found that overexpression of *CD44* in breast cancer and its brain metastasis cohort was associated with poor overall survival. Jakob et al. [46] and others found in their research on squamous cell carcinoma of the head and neck that high expression of *CD44* reduced the overall survival and disease-free survival of patients. Therefore, *LHPP* and *CD44* expression levels may serve as potential biomarkers for predicting the prognosis and immune status of patients with GC. These findings suggest novel targets for future therapeutic strategies.

In summary, *LHPP* plays a key role in the regulation of stemness characteristics and immune microenvironment in GC and may provide new biomarkers and therapeutic targets for personalized treatment of patients with GC. However, this study has certain limitations, including the sample size and need for further clinical validation. Future studies should expand the clinical sample size and explore the specific mechanisms by which *LHPP* regulates the immune microenvironment of GC to promote its translational potential in clinical applications.

## Methods

### Patients and gastric tissue samples

A total of 233 paraffin-embedded tumor tissue microarrays (TMAs) specimens were collected from Fujian Medical University Union Hospital from January 2013 to October 2015. Tissue samples from 141 GC patients, who underwent surgical resection following neoadjuvant chemotherapy, were used for bulk transcriptome sequencing. Tissue samples from 10 GC patients, who underwent surgical resection following neoadjuvant chemotherapy, were used for single-cell transcriptome sequencing. Inclusion criteria were as follows: (1) GC histological identification; (2) availability of follow-up data and clinicopathological features; (3) TNM staging of GC tumors was performed according to the 8th edition of the American Joint Committee on Cancer (AJCC) TNM classification guidelines. Exclusion criteria were as follows: non-formalin-fixed, paraffin-embedded tumor specimens at initial diagnosis, including tumor center (CT) and invasive margin (IM). All procedures performed in studies involving human subjects were in accordance with the Declaration of Helsinki. All patients for whom tissue samples were used in this study provided written informed consent. This study was approved by the Ethics Committee of Fujian Medical University Union Hospital (Ethics Approval number: 2024QH047). All centers approved the study.

### Data collection from public databases

For RNA-seq data from The Cancer Genome Atlas stomach adenocarcinoma (TCGA), fragments per kilobase per million transcripts (FPKM) were converted to transcripts per megabase (TPM) values by the R package “limma”. For microarray data from Affymetrix arrays, we downloaded the company chip raw “CEL” file, corrected it on raw scale, and employed the multiarray averaging method through affy and simpleaffy packages to perform background adjustment and quantile normalization. Taking into account batch effects between datasets, the combatseq function in the SVA software package was used to remove batch effects in different datasets for data normalization.

### Evaluation of immunological characteristics of the tumor microenvironment

We used CIBERSORT, EPIC, MCPcounter, QuanTIseq, TIMER, and Xcell to calculate the infiltrating abundance of immune cells in GC. The tumor immune dysfunction score (TIDE) (http://tide.dfci.harvard.edu/login/) was used to predict the response of patients with GC to immunotherapy.

### Identification of differentially expressed RNA

The empirical Bayesian approach of R package “limma” was used to identify differentially expressed genes (DEGs) for each modification pattern. An adjusted *p* value < 0.05 and an absolute fold change >2 were used as the criteria for the significance of DEGs.

### Gene set enrichment analysis

Gene set enrichment analysis (GSEA) was performed using the molecular Signature database (MSigDB) to identify significantly enriched pathways among different tumor sample groups. A gene set is considered “enriched” if its enrichment score is positive, the expression level of most members of the gene set is high and the risk score is also high. Pathways with a false discovery rate (FDR) adjusted *p* < 0.05 were considered significantly enriched.

### Analysis of mutation and copy number difference

Waterfall plots of gene mutations and copy number variations in the TCGA-STAD cohort were plotted using the R package “maftools”. To analyze copy number, we used the GISTIC 2.0 definition to identify amplified genomes and missing gene sequences. Copy number gain or loss was determined by the total number of genes with altered copy number at the lesion and arm level. Using genpattern website (https://cloud.genepattern.org/) gistic2 plug-in copy number analysis, and using the hg38 human genome sequence as the reference set.

### Correlation analysis of drug sensitivity

The R package “pRRophetic” and “oncoPredict” was used for prediction, and the “linearRidge” function in the R package “ridge” was used to construct a ridge regression model to estimate the IC_50_ of patients with GC to commonly used chemotherapy drugs.

### Immunohistochemistry and evaluation

Serial sections of FFPE samples were 4 μm in size and mounted on glass slides for IHC analysis. Sections were deparaffinized with xylene and rehydrated with alcohol. We blocked endogenous peroxidase by immersing the sections in 3% H2O2 aqueous solution for 10 min and then microwave the sections in 0.01 mol/L sodium citrate buffer, pH 6.0, for 10 min for antigen recovery. The slides were then washed with phosphate buffered saline (PBS) and incubated with 10% normal goat serum (Zhongshan Biotechnology Co., LTD., China) to eliminate nonspecific reactions. Subsequently, sections were incubated with primary antibodies overnight at 4 °C. Negative controls were treated in the same way, but the primary antibody was omitted. After rinsing three times with PBS, secondary antibodies were diluted, incubated on slides for 30 min at room temperature, and stained with diamine benzidine (DAB) solution. Finally, the slides were counterstained with heme, dehydrated, and fixed with cover glass and neutral resin.

For staining of LHPP and CD44, the H-score was quantified using: H-score = (1 × % weak staining) + (2 × % medium staining) + (3 × % strong staining).

To assess immune cell infiltration, five representative and independent fields were captured at ×200 magnification at the tumor center (CT) and the invasive margin (IM), as shown in Fig. 7E. Next, we assisted label counting using the “Measure” plug-in in the Image-Pro Plus software to obtain the number of positive cells in the field. The average number of positive cells in the five field areas was divided by the field area (0.27 mm^2^) to obtain the infiltration density of immune cells in CT and IM. The percentage/number of all positive cells is expressed as the mean of five randomly selected microscopy fields.

Inflammation, exclusion, and desert phenotypes were determined based on immunocytochemical staining slides for CD8^+^, and the three immunophenotypes were classified based on features reported in previous studies, as shown in Fig. 7E.

Information and concentrations of reagents used for IHC are provided below: LHPP (15759-1-AP, Proteintech, 1:500), CD44 (ET1609-74, HUABIO, 1:1000), CD4 (ET1609-52, HUABIO, 1:800), CD45 (ET7111-03, HUABIO, 1:1000), CD3 (HA720082, HUABIO, 1:1000), CD8 (ET1606-31, HUABIO, 1:200), CD45RO (ab23, Abcam, 1:800), FOXP3 (ET1702-12, HUABIO, 1:200).

The IHC results were evaluated by two independent gastroenterology pathologists who were blinded to the clinical prognosis of the patients. Approximately 90% of the scoring results were the same. When the scores of the two independent pathologists diverged, another pathologist checked the results again and selected one of the scores proposed by the first two doctors, or the three pathologists discussed the decision together.

### Preparation and processing of single-cell RNA sequencing data

Single-cell gel bead emulsions were generated from single-cell suspensions using a 10×Genomics Chromium Controller. cDNA was obtained from mRNA by dribble and amplified by reverse transcription reaction according to the manufacturer’s instructions. Te 10× libraries were sequenced on a NovaSeq sequencing platform (Illumina, San Diego, CA). CellRanger (version 4.0.0) was used to obtain fastq files of the raw data and annotated with the human genome reference sequence (GRCh38). Gene barcoding matrices were then obtained following the Seurat (version 4.0.4) pipeline in R software (version 4.0.5, R-Foundation, Vienna, Austria). Cells with a detected gene number below 250 or above 4000, or a high ratio of mitochondrial transcripts (more than 20%), were not included in the analysis. Following normalization and scaling, the harmony algorithm was used to remove batch effects between patients. The top 2000 highly variable genes were selected for principal component analysis (PCA) method and the top 20 principal components (PCs) were used for cluster analysis. To identify differentially expressed marker genes for each cell type, the FindAllMarkers function in Seurat was used under default parameters. Marker genes were selected as those with adjusted *p* values less than 0.05, average logFC larger than 1, and percentage of cells with expression higher than 0.25.

### Markov Affinity-based Graph Imputation of Cells (MAGIC) for denoising and imputing single-cell RNA sequencing data

Markov Affinity-based Graph Imputation of Cells (MAGIC) is a denoising algorithm for high-dimensional data, widely used in single-cell RNA sequencing [47]. In this study, MAGIC was applied to remove technical noise from single-cell RNA sequencing data and restore the underlying biological structure. First, the data was normalized by dividing the gene expression of each cell by the total expression and applying a logarithmic transformation. MAGIC then constructs a similarity graph between cells, calculates the similarity in gene expression patterns, and selects the top K nearest cells as neighbors, using a Gaussian kernel function to compute the similarity. Next, MAGIC uses a Markov chain to smooth the graph by iteratively updating the gene expression values of the cells, allowing them to be influenced by the expression values of their neighbors, thereby reducing noise and imputing missing data. In our analysis, we used 7586 epithelial cells, set K = 10 to determine the 10 most similar neighbors for each cell, and set the number of steps to *t* = 5. Through this process, MAGIC effectively reduces noise, recovers the underlying structure of the data, and highlights the biological signals in cell gene expression.

### Cell culture and reagents

All human GC cell lines were purchased from Guangzhou Cellcook Biotech Co., Ltd (Guangzhou, China). The mouse GC cell line YTN2, YTN3, YTN5 and YTN16 were a generous gift from Dr. Sachiyo Nomura, University of Tokyo. AGS was cultured in Ham’s F-12 Nutrient Mixture (GIBCO, Carlsbad, CA) containing 1% penicillin/streptomycin (GIBCO) and 10% fetal bovine serum (Invitrogen Life Technologies, Carlsbad, CA). HGC-27, SNU-216, MKN28, MKN45, KATO III and NCI-N87 were maintained in RPMI/1640 medium with 10% fetal bovine serum and 1% penicillin/streptomycin. YTN2, YTN3, YTN5 and YTN16 were maintained in Dulbecco’s modified Eagle’s medium (DMEM, D6429, Sigma-Aldrich) with 10% fetal bovine serum, 1% penicillin/streptomycin and MITO+ serum extender (No. 355006, Thomas Scientific). All these cell lines were cultured at 37 °C in a humidified incubator with 5% CO2. Mycoplasma infection was routinely examined once a month.

### Tumor spheroid culture

Fetal bovine serum (FBS) was obtained from Umedium HeFei China. Cells were seeded into ultra-low attachment 6-well dishes (Corning Life Sciences, NY, USA) and cultured in Ham’s F-12K (Kaighn’s) containing 20 ng/ml epidermal growth factor (EGF), 10 ng/ml basic fibroblast growth factor (bFGF), 2% B-27 (Life Technologies, Gaithersburg, MD, USA), and 2 mM L-glutamine (Life Technologies, Gaithersburg, MD, USA) as previously described. Spheroids were incubated in a 5% CO_2_ chamber at 37 °C for seven days. The culture medium was changed every three days. The diameter and number of tumor spheres in three random magnification fields were calculated under All-in-one Fluorescence Microscope (BZ-X700, Keyence Corp, Atlanta, GA, USA) in the bright light model. Spheroids were collected after 7 days except when noted otherwise. Protein was extracted for analysis, or cells were dissociated with Accutase (Innovative Cell Technologies, San Diego, CA, USA) and used for other experiments.

### Western blot analysis

RIPA buffer (middle) (Shanghai Beyotime, China) is used to lyse tissues and cells. 40 μg of protein was loaded into each pore, separated by 10% SDS-PAGE, and subsequently transferred onto a polyvinylidene fluoride membrane (EMD Millipore, Billerica, MA, USA). Following blocking, the membrane was incubated with primary antibody overnight in a dilution buffer specifically designed for primary antibodies (Thermo Fisher Scientific, Shanghai, China). Following TBST washing, the membrane was subjected to incubation with corresponding antibodies at room temperature for 1 h and subsequently washed thrice with TBST. The proteins on the membranes were visualized using enhanced chemiluminescence (Amersham; GE Healthcare). Experiments were performed in triplicate. Utilize the ImageJ software for the analysis of grayscale values in each strip. Each experimental group is compared to the control group as a reference, and the relative multiplication relationship is subsequently computed.

Information and concentrations of reagents used for Western blot are provided below: LHPP (15759-1-AP, Proteintech, 1:500), beta Tubulin (EM0103, HUABIO, 1:10,000), CD44 (ET1609-74, HUABIO, 1:1000), NANOG (ET1610-2, HUABIO, 1:2000), SOX2 (R1106-1, HUABIO, 1:1000), SOX9 (ET1611-56, HUABIO, 1:2000), GSK3 beta (ET1607-71, HUABIO, 1:1000), Phospho-GSK3 beta (ET1607-60, HUABIO, 1:1000).

### Mice

*Mist1-CreERT2* (Cat# 029228), *Rosa26-LSL-Tdtomato* (Cat# 007914), *Apc*^*fl/fl*^ (Cat# 029275) and *p53*^*fl/fl*^ (Cat# 008462) mice were purchased from the Jackson Laboratory (Bar Harbor, Maine, USA). The primers used for genotyping in this study are listed in Table S3. Wild-type C57BL/6 mice were purchased from Shanghai Slac Laboratory Animal Co., Ltd (Shanghai, China). All mice used were 6–8 weeks of age. All animal experiments were performed according to the Animal Protection Committee of Fujian Medical University (Fuzhou, China) and approved by the Ethics Committee of Fujian Medical University/Laboratory Animal Center (Fuzhou, China).

### Tumor xenograft assay

All BALB/c nude mice (4–6 weeks of age) used in our study were purchased from Beijing Vital River Laboratory Animal Technology Co., Ltd (Beijing, China). Wild-type C57BL/6 mice (6–8 weeks of age) were purchased from Shanghai Slac Laboratory Animal Co., Ltd (Shanghai, China). To evaluate the impact of LHPP on stemness, limiting dilution assays were performed in nude mice and Wild-type C57BL/6 mice. Cells were injected subcutaneously into the right axillary fossa of nude mice at indicated cell concentrations. Five mice were used in each experimental group. Tumor formation was checked every 3–4 days and the mice were sacrificed at 4–6 weeks after injection and the tumors were weighed and used in immunohistochemical staining studies. Tumor volume was calculated with the following formula: V = (L × W^2^)/2 (V, tumor volume; L, length; W, width), and growth curves were plotted using average tumor volume within each experimental group at the set time points. The frequency of tumor-initiating cells was calculated using the extreme limiting dilution analysis program (http://bioinf.wehi.edu.au/software/elda/).

### Organoid culture

Human organoids culture was performed following previously published protocol. Briefly, tumor tissues from the stomach were washed with PBS containing 1× Penicillin/Streptomycin twice (BL505A, Biosharp, Hefei, China), followed by removing the muscle layer and mucus using scissors. Subsequently, the sample should be sliced into 2–3 mm sections and subjected to enzymatic digestion using 2.5 mg/ml of Collagenase A. (Sigma-Aldrich, St. Louis, MO, USA) for 30 min. Five ml Dissociation buffer, containing D-sorbitol (Sigma-Aldrich, St. Louis, MO, USA) and sucrose (Sigma-Aldrich, St. Louis, MO, USA), was added to the tissue and agitated for 2 min. The final supernatant was filtered through a 70 μm mesh and the crypts fraction was centrifuged at 150 g for 5 min. After being washed with ice-cold PBS, the gland pellet was resuspended in Matrigel™ (356255, Corning, USA) supplemented with standard gastric organoid advanced DMEM/F12 (#12634010, Thermo Fisher Scientific, Waltham, MA, USA), 1× GlutaMax (#35050061, Thermo Fisher Scientific, Waltham, MA, USA), 1× HEPES (#15630080, Thermo Fisher Scientific, Waltham, MA, USA), 1× Penicillin/Streptomycin, 50% Wnt3a, 10% RSPO-1, 10% Noggin, 1× B27 (#17504001, Thermo Fisher Scientific, Waltham, MA, USA), 50 ng/mL EGF (PHG0311, Thermo Fisher Scientific, Waltham, MA, USA), 200 ng/mL FGF10 (#100-26, Peprotech, Rocky Hill, NJ, USA), 1 mM N-acetyl-L-cysteine (#A9165, Sigma-Aldrich, St. Louis, MO, USA), 1 nM Gastrin (#G9145, Sigma-Aldrich, St. Louis, MO, USA), 2 mM A83-01 (#2939/10, Tocris, Bristol, UK), 10 mM Y-27632 (#1254/10, Tocris Bristol, UK). Finally, 50 μl Matrigel™ suspension was carefully ejected into the center of each well of the 24-well plate. 1 ml of standard gastric organoid medium was added to each well. The organoids were cultured in a 5% CO_2_ incubator at 37 °C and the media was changed every 2–3 days. Organoids from the second passage were infected with lentivirus carrying either control or RPRD1A overexpression in 15 ml tubes overnight. Seven days after infection, the diameter and number of organoids were measured under a light microscope in three random fields magnified at 100×. For histological examination of the organoids, they were fixed in 4% paraformaldehyde for 1 h and subsequently embedded in a 2% agarose gel or directly fixed in formalin-containing Matrigel to generate paraffin blocks for sectioning and staining.

### Flow cytometry assay

The stably transfected GC cells were digested and centrifuged and placed in 1.5 ml tubes. The cells were washed three times with PBS and centrifuged at 1000 rpm for 5 min. This supernatant was discarded and subsequently added to 100 μl staining buffer (PBS, pH 7.4, 0.1%BSA) containing 1 μg/ml CD44 antibody and incubated at 4 °C for 30 min. The cells were subsequently resuspended in PBS without undergoing washing and subjected to collection on a FACS flow cytometer as per the manufacturer’s instructions. The results obtained were analyzed using FlowJo software.

### Statistical analysis

All data were processed using SPSS 25.0 (SPSS Inc. Chicago, IL) and R software (version 4.0.0). Student’s *t* test or Wilcoxon rank-sum test was used for continuous variables. We used the *χ*² test or Fisher exact test to compare categorical variables of clinical characteristics. The Kaplan–Meier method was used to estimate median survival. The log-rank test was used to compare survival between two groups. The association of relevant clinicopathological variables with overall survival was assessed using the Cox proportional hazard model. Interactions between the clinicopathological parameters and responsiveness to chemotherapy were tested with the Cox model. Clustering charts based on the *Z*-score normalization method were used to describe the level of the expression in each case. We defined the survival time of patients who were lost to follow-up as the time from surgery to the last follow-up time, and the survival time of patients who were still alive at the end of the study was defined as the time from surgery to the database deadline. Two-tailed *p* values < 0.05 were indicated significant differences.

## Supplementary information

Figure S1-S18, Table S1-S3

Supplementary Figure Legends

Full length western blots

## Data Availability

The sequencing data generated in this study came from GEO database and TCGA database. The dataset analyzed for this study is available from the corresponding author upon reasonable request.
