# Supplementary material for: Targeting LHPP in neoadjuvant chemotherapy resistance of gastric cancer: insights from single-cell and multi-omics data on tumor immune microenvironment and stemness characteristics
Source: Cell Death Dis. 2025 Apr 16;16(1):306. doi: 10.1038/s41419-025-07614-z (PMC12003742; doi:10.1038/s41419-025-07614-z)
Supplement: Supplementary file 1 — Figure S1-S18, Table S1-S3 [file 41419_2025_7614_MOESM1_ESM.pdf]

Figure S1

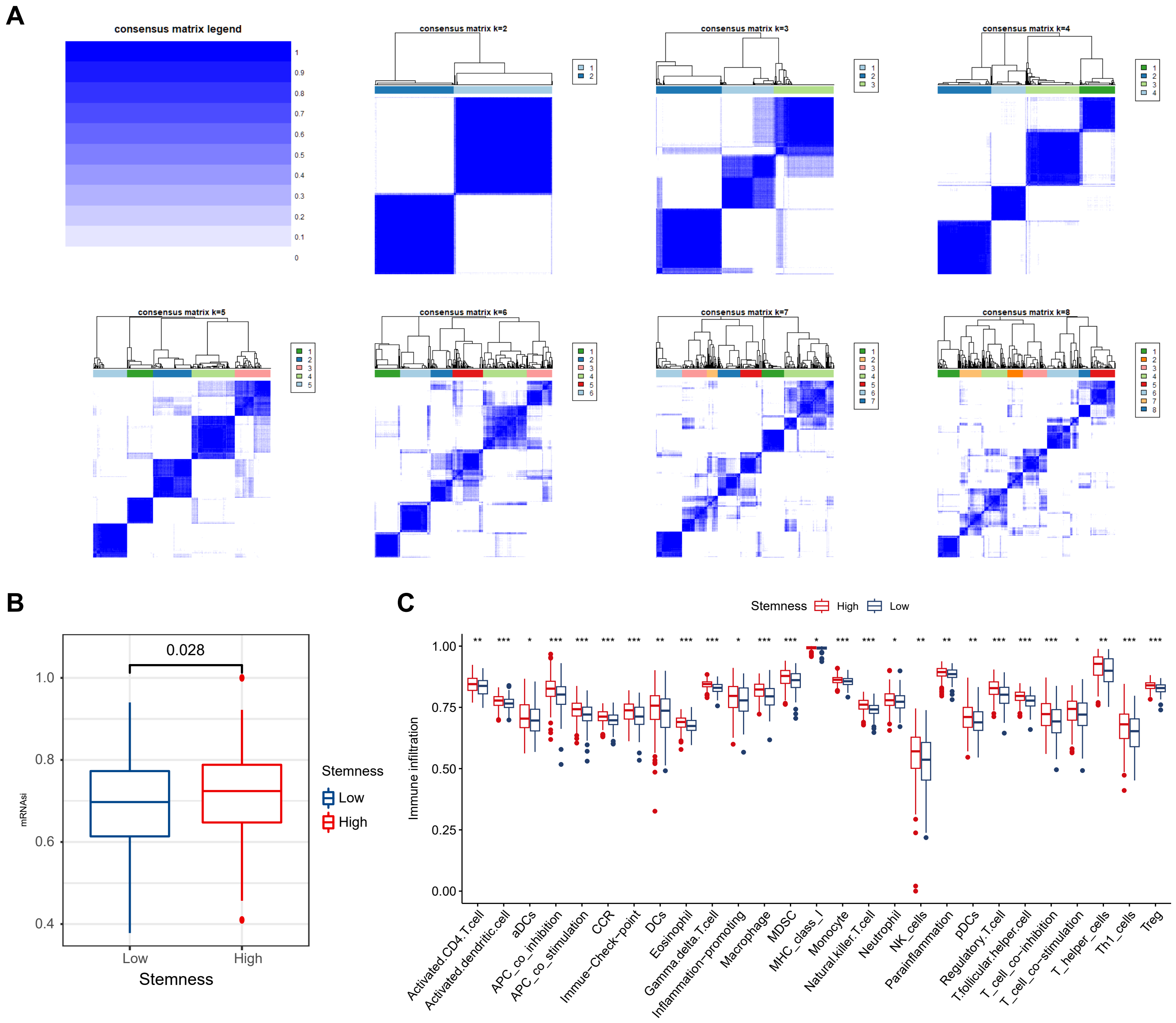
A
consensus matrix legend
consensus matrix k=2
consensus matrix k=3
consensus matrix k=4
consensus matrix k=5
consensus matrix k=6
consensus matrix k=7
consensus matrix k=8
B
0.028
mRNAsi
Stemness
Low
High
C
Stemness High Low
Immune infiltration
Activated.CD4.T.cell
Activated.dendritic.cell
aDCs
APC_co_inhibition
APC_co_stimulation
CCR
Immue−Check−point
DCs
Eosinophil
Gamma.delta.T.cell
Inflammation−promoting
Macrophage
MDSC
MHC_class_I
Monocyte
Natural.killer.T.cell
Neutrophil
NK_cells
Parainflammation
pDCs
Regulatory.T.cell
T.follicular.helper.cell
T_cell_co−inhibition
T_cell_co−stimulation
T_helper_cells
Th1_cells
Treg

Figure S2

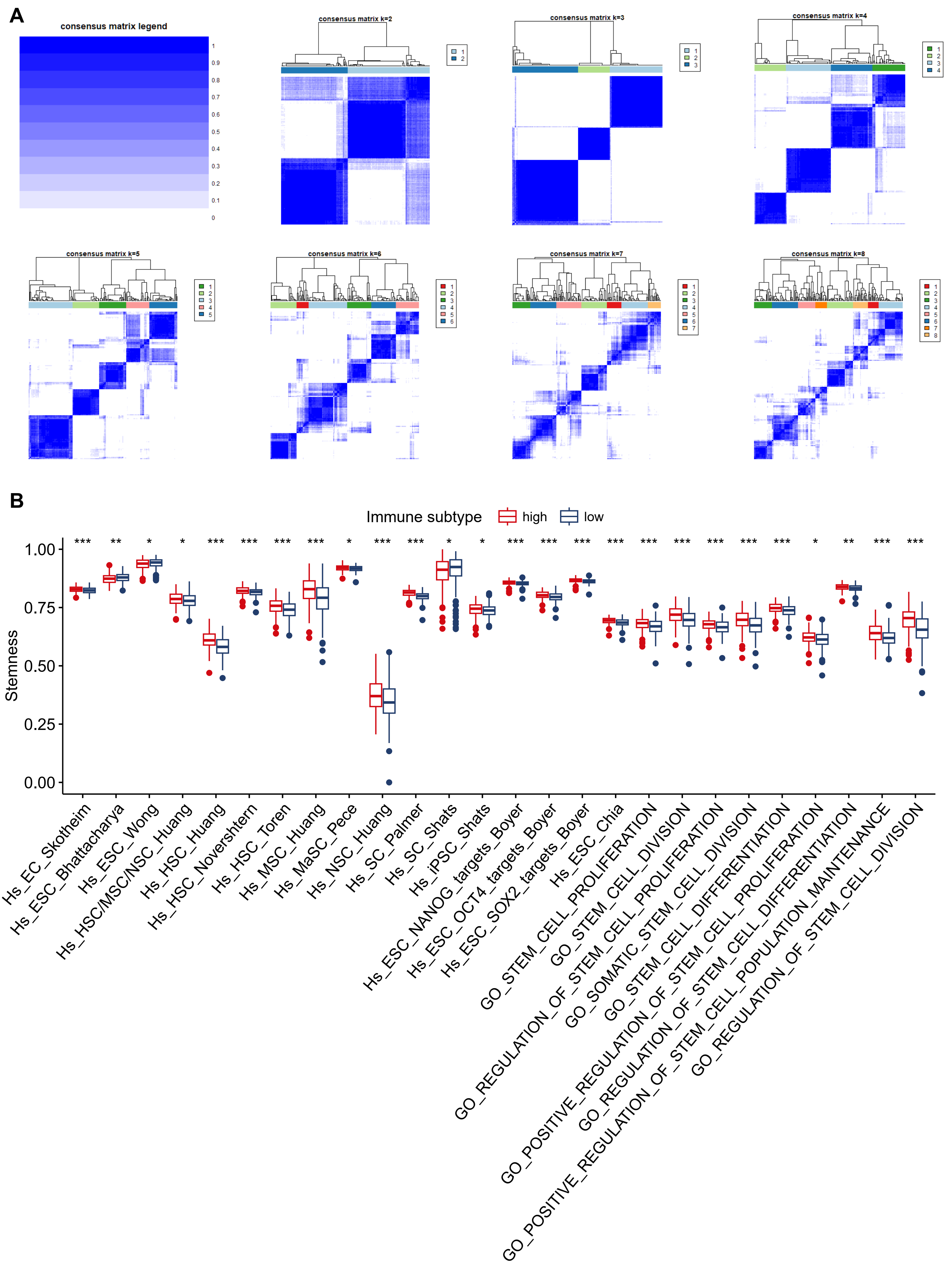
A
consensus matrix legend
consensus matrix k=2
consensus matrix k=3
consensus matrix k=4
consensus matrix k=5
consensus matrix k=6
consensus matrix k=7
consensus matrix k=8
B
Immune subtype high low
Stemness
1.00
0.75
0.50
0.25
0.00
Hs_EC_Skotheim
Hs_ESC_Bhattacharya
Hs_ESC_Wong
Hs_HSC/MSC/NSC_Huang
Hs_HSC_Huang
Hs_HSC_Novershtern
Hs_HSC_Toren
Hs_MSC_Huang
Hs_MaSC_Pece
Hs_NSC_Huang
Hs_SC_Palmer
Hs_SC_Shats
Hs_iPSC_Shats
Hs_ESC_NANOG_targets_Boyer
Hs_ESC_OCT4_targets_Boyer
Hs_ESC_SOX2_targets_Boyer
Hs_ESC_Chia
GO_STEM_CELL_PROLIFERATION
GO_STEM_CELL_DIVISION
GO_REGULATION_OF_STEM_CELL_PROLIFERATION
GO_SOMATIC_STEM_CELL_DIVISION
GO_STEM_CELL_DIFFERENTIATION
GO_POSITIVE_REGULATION_OF_STEM_CELL_PROLIFERATION
GO_REGULATION_OF_STEM_CELL_DIFFERENTIATION
GO_STEM_CELL_POPULATION_MAINTENANCE
GO_POSITIVE_REGULATION_OF_STEM_CELL_DIVISION

Figure S3

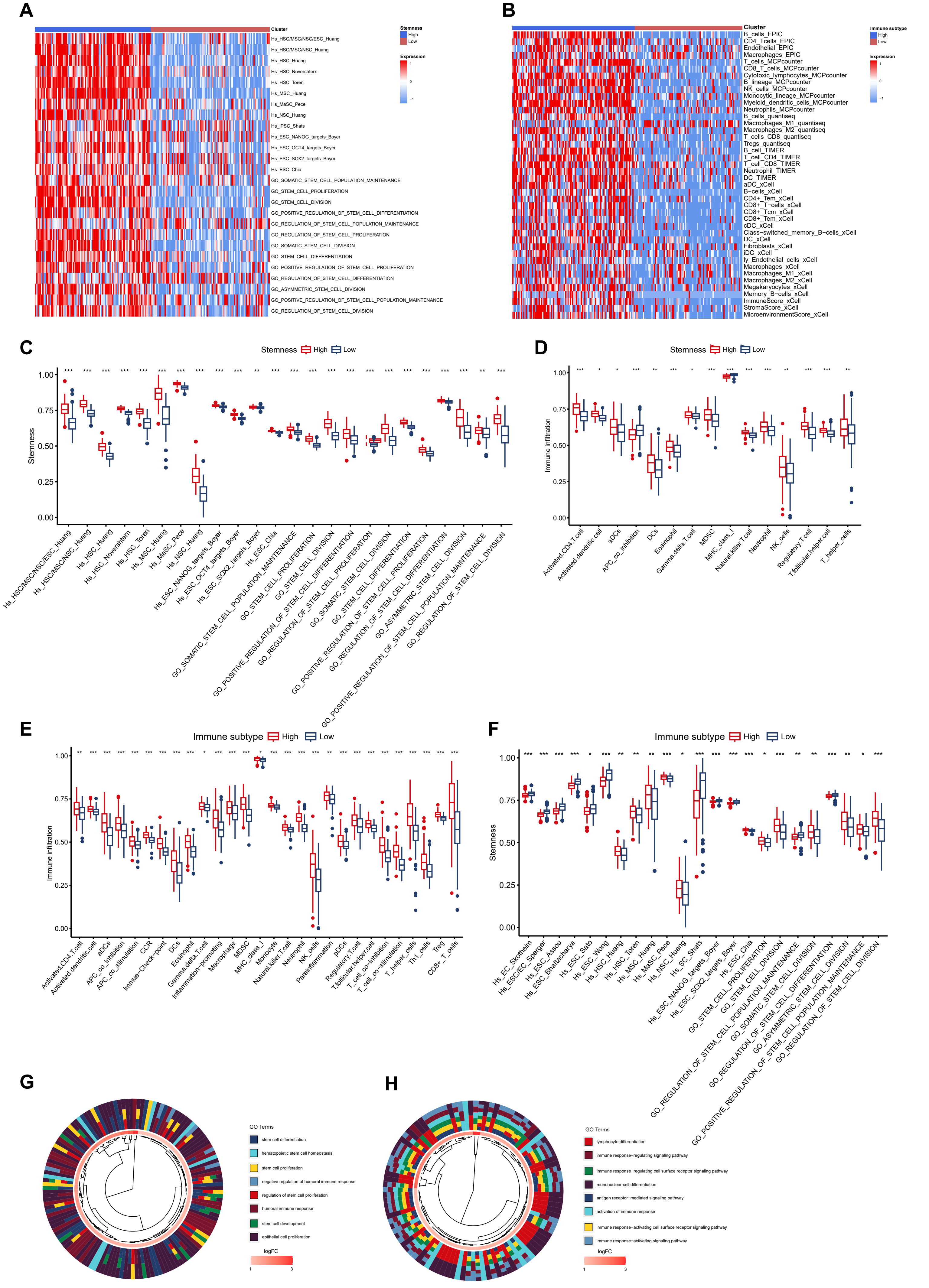

A
Cluster
Stemness
High
Low
Expression
Hs_HSC/MSC/NSC/ESC_Huang
Hs_HSC/MSC/NSC_Huang
Hs_HSC_Huang
Hs_HSC_Novershtern
Hs_HSC_Toren
Hs_MSC_Huang
Hs_MaSC_Pece
Hs_NSC_Huang
Hs_iPSC_Shats
Hs_ESC_NANOG_targets_Boyer
Hs_ESC_OCT4_targets_Boyer
Hs_ESC_SOX2_targets_Boyer
Hs_ESC_Chia
GO_SOMATIC_STEM_CELL_POPULATION_MAINTENANCE
GO_STEM_CELL_PROLIFERATION
GO_STEM_CELL_DIVISION
GO_POSITIVE_REGULATION_OF_STEM_CELL_DIFFERENTIATION
GO_REGULATION_OF_STEM_CELL_POPULATION_MAINTENANCE
GO_REGULATION_OF_STEM_CELL_PROLIFERATION
GO_SOMATIC_STEM_CELL_DIVISION
GO_STEM_CELL_DIFFERENTIATION
GO_POSITIVE_REGULATION_OF_STEM_CELL_PROLIFERATION
GO_REGULATION_OF_STEM_CELL_DIFFERENTIATION
GO_ASYMMETRIC_STEM_CELL_DIVISION
GO_POSITIVE_REGULATION_OF_STEM_CELL_POPULATION_MAINTENANCE
GO_REGULATION_OF_STEM_CELL_DIVISION
B
Cluster
Immune subtype
High
Low
Expression
B_cells_EPIC
CD4_Tcells_EPIC
Endothelial_EPIC
Macrophages_EPIC
T_cells_MCPcounter
CD8_T_cells_MCPcounter
Cytotoxic_lymphocytes_MCPcounter
B_lineage_MCPcounter
NK_cells_MCPcounter
Monocytic_lineage_MCPcounter
Myeloid_dendritic_cells_MCPcounter
Neutrophils_MCPcounter
B_cells_quantiseq
Macrophages_M1_quantiseq
Macrophages_M2_quantiseq
T_cells_CD8_quantiseq
Tregs_quantiseq
B_cell_TIMER
T_cell_CD4_TIMER
T_cell_CD8_TIMER
Neutrophil_TIMER
DC_TIMER
aDC_xCell
B-cells_xCell
CD4+_Tem_xCell
CD8+_T-cells_xCell
CD8+_Tcm_xCell
CD8+_Tem_xCell
cDC_xCell
Class-switched_memory_B-cells_xCell
DC_xCell
Fibroblasts_xCell
iDC_xCell
ly_Endothelial_cells_xCell
Macrophages_xCell
Macrophages_M1_xCell
Macrophages_M2_xCell
Megakaryocytes_xCell
Memory_B-cells_xCell
ImmuneScore_xCell
StromaScore_xCell
MicroenvironmentScore_xCell
C
Stemness High Low
Stemness
1.00
0.75
0.50
0.25
0.00
D
Stemness High Low
Immune infiltration
Activated.CD4.T.cell
Activated.dendritic.cell
aDCs
APC_co_inhibition
DCs
Eosinophil
Gamma.delta.T.cell
MDSC
MHC_class_I
Natural.killer.T.cell
Neutrophil
NK_cells
Regulatory.T.cell
T.follicular.helper.cell
T_helper_cells
E
Immune subtype High Low
Immune infiltration
Activated.CD4.T.cell
Activated.dendritic.cell
aDCs
APC_co_inhibition
APC_co_stimulation
CCR
Immue-Check-point
DCs
Eosinophil
Gamma.delta.T.cell
Inflammation-promoting
Macrophage
MDSC
MHC_class_I
Monocyte
Natural.killer.T.cell
Neutrophil
NK_cells
Parainflammation
pDCs
Regulatory.T.cell
T.follicular.helper.cell
T_cell_co-inhibition
T_cell_co-stimulation
T_helper_cells
Th1_cells
Treg
CD8+_T_cells
F
Immune subtype High Low
Stemness
Hs_EC_Skotheim
Hs_ESC/EC_Sperger
Hs_ESC_Assou
Hs_ESC_Bhattacharya
Hs_ESC_Sato
Hs_ESC_Wong
Hs_HSC_Huang
Hs_HSC_Toren
Hs_MSC_Huang
Hs_MaSC_Pece
Hs_NSC_Huang
Hs_SC_Shats
Hs_ESC_NANOG_targets_Boyer
Hs_ESC_SOX2_targets_Boyer
Hs_ESC_Chia
GO_STEM_CELL_PROLIFERATION
GO_STEM_CELL_DIVISION
GO_REGULATION_OF_STEM_CELL_POPULATION_MAINTENANCE
GO_SOMATIC_STEM_CELL_DIVISION
GO_REGULATION_OF_STEM_CELL_DIFFERENTIATION
GO_ASYMMETRIC_STEM_CELL_DIVISION
GO_POSITIVE_REGULATION_OF_STEM_CELL_POPULATION_MAINTENANCE
GO_REGULATION_OF_STEM_CELL_DIVISION
G
GO Terms
stem cell differentiation
hematopoietic stem cell homeostasis
stem cell proliferation
negative regulation of humoral immune response
regulation of stem cell proliferation
humoral immune response
stem cell development
epithelial cell proliferation
logFC
1
3
H
GO Terms
lymphocyte differentiation
immune response−regulating signaling pathway
immune response−regulating cell surface receptor signaling pathway
mononuclear cell differentiation
antigen receptor−mediated signaling pathway
activation of immune response
immune response−activating cell surface receptor signaling pathway
immune response−activating signaling pathway
logFC
1
3

Figure S4

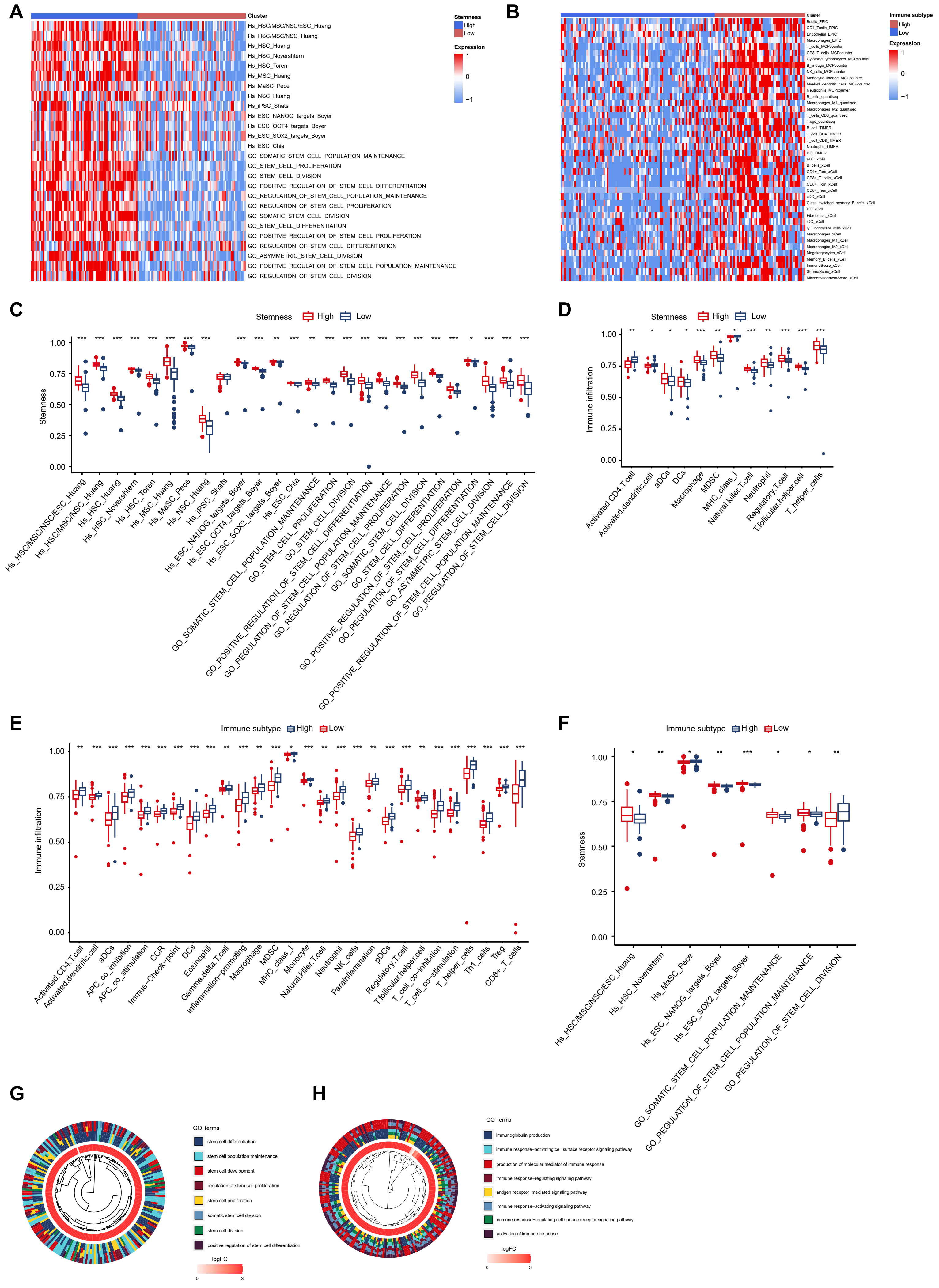
A
Cluster
Hs_HSC/MSC/NSC/ESC_Huang
Hs_HSC/MSC/NSC_Huang
Hs_HSC_Huang
Hs_HSC_Novershtern
Hs_HSC_Toren
Hs_MSC_Huang
Hs_MaSC_Pece
Hs_NSC_Huang
Hs_iPSC_Shats
Hs_ESC_NANOG_targets_Boyer
Hs_ESC_OCT4_targets_Boyer
Hs_ESC_SOX2_targets_Boyer
Hs_ESC_Chia
GO_SOMATIC_STEM_CELL_POPULATION_MAINTENANCE
GO_STEM_CELL_PROLIFERATION
GO_STEM_CELL_DIVISION
GO_POSITIVE_REGULATION_OF_STEM_CELL_DIFFERENTIATION
GO_REGULATION_OF_STEM_CELL_POPULATION_MAINTENANCE
GO_REGULATION_OF_STEM_CELL_PROLIFERATION
GO_SOMATIC_STEM_CELL_DIVISION
GO_STEM_CELL_DIFFERENTIATION
GO_POSITIVE_REGULATION_OF_STEM_CELL_PROLIFERATION
GO_REGULATION_OF_STEM_CELL_DIFFERENTIATION
GO_ASYMMETRIC_STEM_CELL_DIVISION
GO_POSITIVE_REGULATION_OF_STEM_CELL_POPULATION_MAINTENANCE
GO_REGULATION_OF_STEM_CELL_DIVISION
Stemness
High
Low
Expression
1
0
−1
B
Immune subtype
High
Low
Expression
1
0
−1
C
Stemness
High
Low
Stemness
1.00
0.75
0.50
0.25
0.00
D
Stemness
High
Low
Immune infiltration
Activated.CD4.T.cell
Activated.dendritic.cell
aDCs
DCs
Macrophage
MDSC
MHC_class_I
Natural.killer.T.cell
Neutrophil
Regulatory.T.cell
T.follicular.helper.cell
T_helper_cells
E
Immune subtype
High
Low
Immune infiltration
Activated.CD4.T.cell
Activated.dendritic.cell
aDCs
APC_co_inhibition
APC_co_stimulation
CCR
Immune−Check−point
DCs
Eosinophil
Gamma.delta.T.cell
Inflammation−promoting
Macrophage
MDSC
MHC_class_I
Monocyte
Natural.killer.T.cell
Neutrophil
NK_cells
Parainflammation
pDCs
Regulatory.T.cell
T.follicular.helper.cell
T_cell_co−inhibition
T_cell_co−stimulation
T_helper_cells
Th1_cells
Treg
CD8+_T_cells
F
Immune subtype
High
Low
Stemness
Hs_HSC/MSC/NSC/ESC_Huang
Hs_HSC_Novershtern
Hs_MaSC_Pece
Hs_ESC_NANOG_targets_Boyer
Hs_ESC_SOX2_targets_Boyer
GO_SOMATIC_STEM_CELL_POPULATION_MAINTENANCE
GO_REGULATION_OF_STEM_CELL_POPULATION_MAINTENANCE
GO_REGULATION_OF_STEM_CELL_DIVISION
G
GO Terms
stem cell differentiation
stem cell population maintenance
stem cell development
regulation of stem cell proliferation
stem cell proliferation
somatic stem cell division
stem cell division
positive regulation of stem cell differentiation
logFC
0
3
H
GO Terms
immunoglobulin production
immune response−activating cell surface receptor signaling pathway
production of molecular mediator of immune response
immune response−regulating signaling pathway
antigen receptor−mediated signaling pathway
immune response−activating signaling pathway
immune response−regulating cell surface receptor signaling pathway
activation of immune response
logFC
0
3

Figure S5

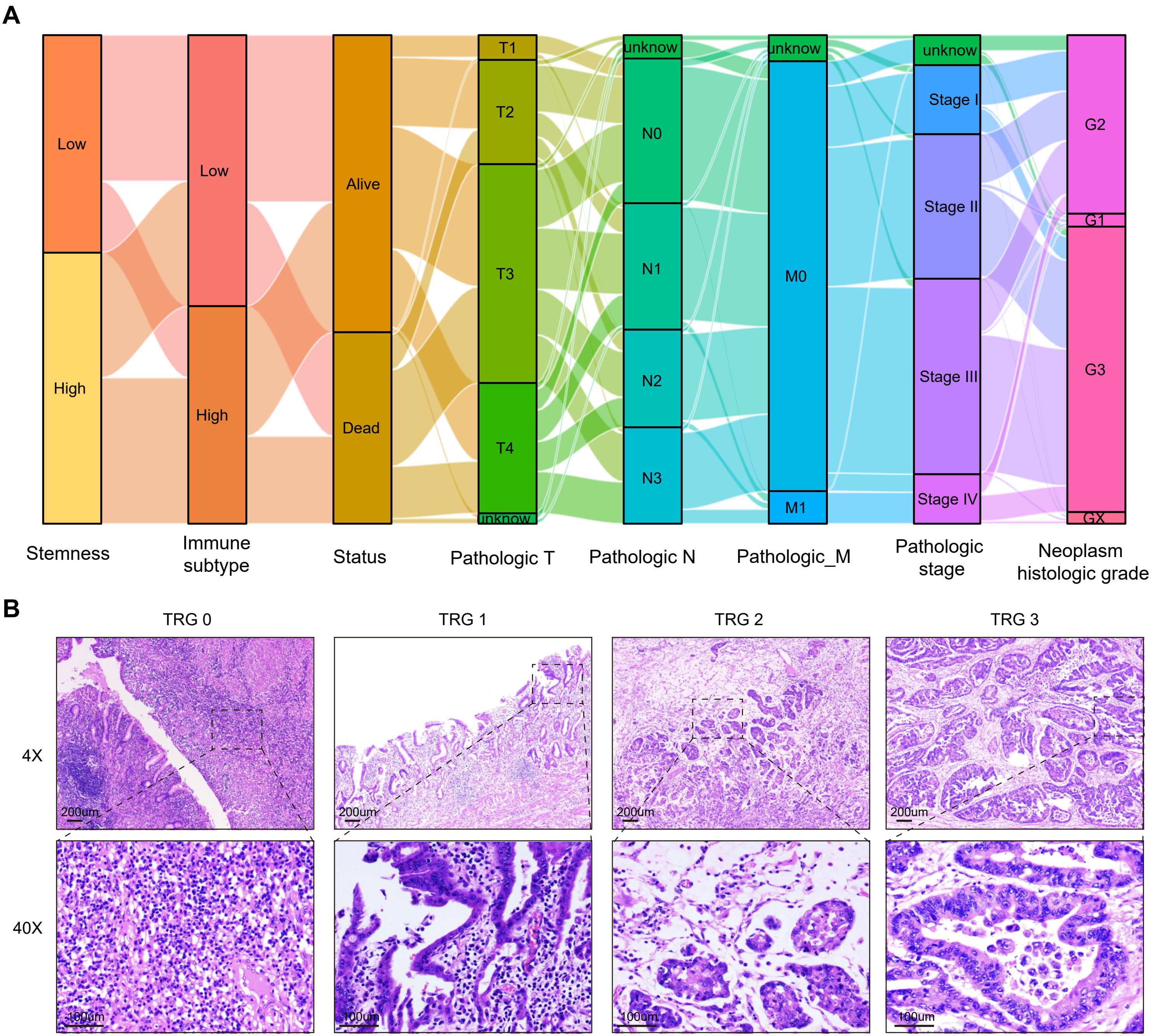

A
Low
High
Low
High
Alive
Dead
T1
T2
T3
T4
unknow
unknow
N0
N1
N2
N3
unknow
M0
M1
unknow
Stage I
Stage II
Stage III
Stage IV
G2
G1
G3
GX
Stemness
Immune subtype
Status
Pathologic T
Pathologic N
Pathologic_M
Pathologic stage
Neoplasm histologic grade
B
TRG 0
TRG 1
TRG 2
TRG 3
4X
40X
200um
200um
200um
200um
100um
100um
100um
100um

Figure S6

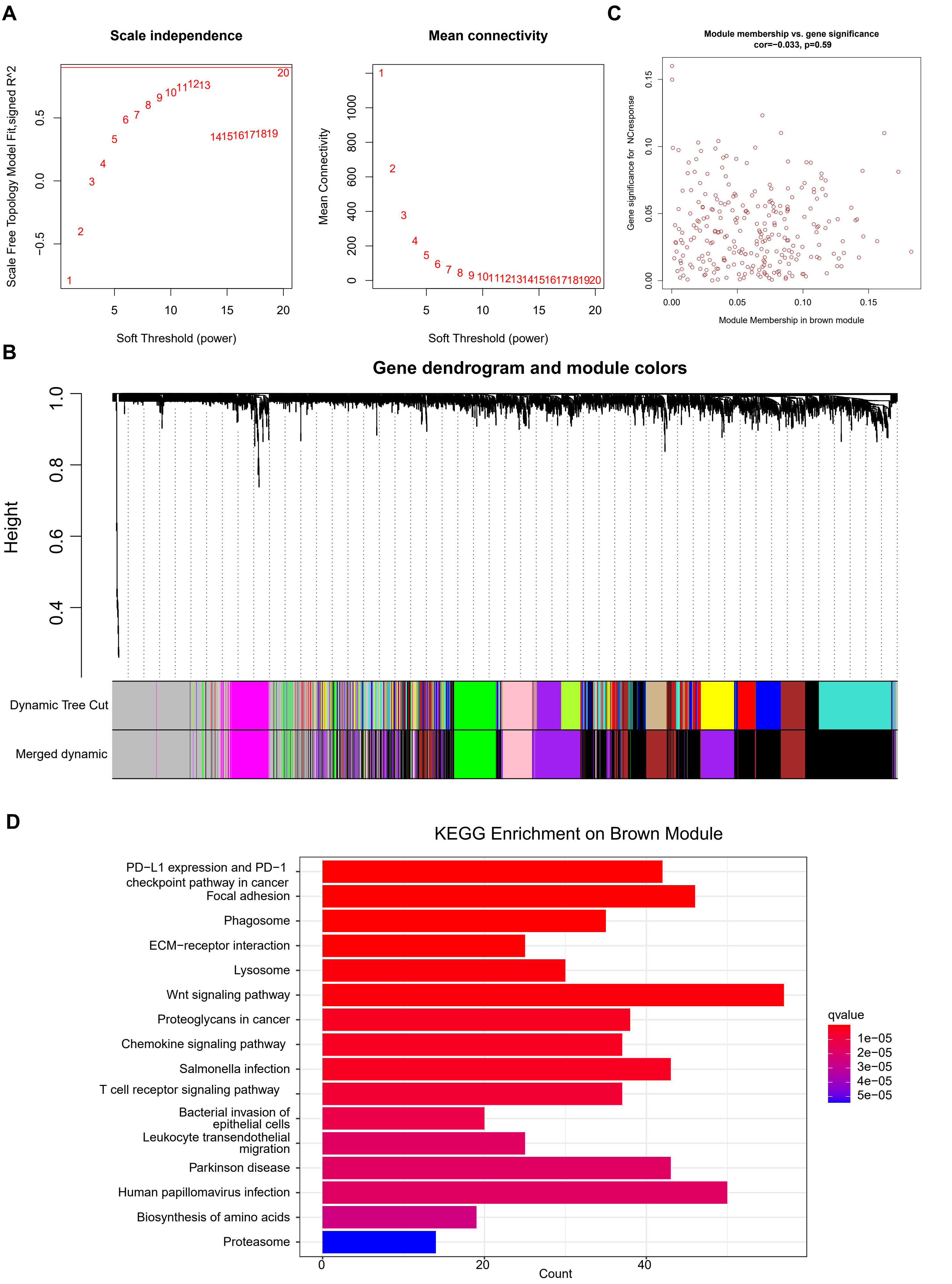
A
Scale independence
Scale Free Topology Model Fit,signed R^2
Soft Threshold (power)
Mean connectivity
Mean Connectivity
Soft Threshold (power)
C
Module membership vs. gene significance
cor=−0.033, p=0.59
Gene significance for NCresponse
Module Membership in brown module
B
Gene dendrogram and module colors
Height
Dynamic Tree Cut
Merged dynamic
D
KEGG Enrichment on Brown Module
PD−L1 expression and PD−1 checkpoint pathway in cancer
Focal adhesion
Phagosome
ECM−receptor interaction
Lysosome
Wnt signaling pathway
Proteoglycans in cancer
Chemokine signaling pathway
Salmonella infection
T cell receptor signaling pathway
Bacterial invasion of epithelial cells
Leukocyte transendothelial migration
Parkinson disease
Human papillomavirus infection
Biosynthesis of amino acids
Proteasome
Count
qvalue
1e−05
2e−05
3e−05
4e−05
5e−05

Figure S7

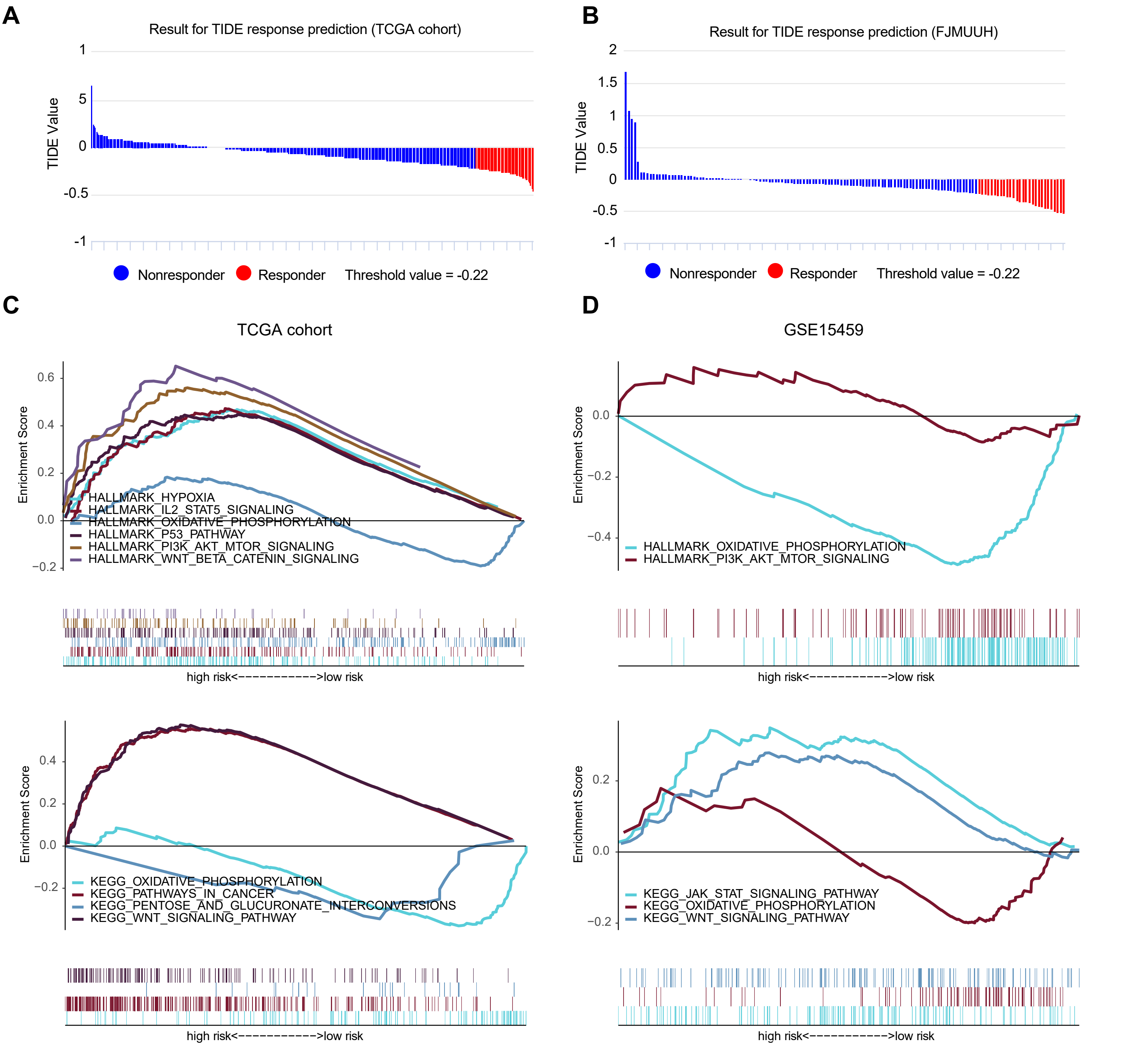

A
Result for TIDE response prediction (TCGA cohort)
TIDE Value
1
5
0
-0.5
-1
Nonresponder
Responder
Threshold value = -0.22
B
Result for TIDE response prediction (FJMUUH)
TIDE Value
2
1.5
1
0.5
0
-0.5
-1
Nonresponder
Responder
Threshold value = -0.22
C
TCGA cohort
Enrichment Score
HALLMARK_HYPOXIA
HALLMARK_IL2_STAT5_SIGNALING
HALLMARK_OXIDATIVE_PHOSPHORYLATION
HALLMARK_P53_PATHWAY
HALLMARK_PI3K_AKT_MTOR_SIGNALING
HALLMARK_WNT_BETA_CATENIN_SIGNALING
high risk<----------->low risk
KEGG_OXIDATIVE_PHOSPHORYLATION
KEGG_PATHWAYS_IN_CANCER
KEGG_PENTOSE_AND_GLUCURONATE_INTERCONVERSIONS
KEGG_WNT_SIGNALING_PATHWAY
high risk<----------->low risk
D
GSE15459
Enrichment Score
HALLMARK_OXIDATIVE_PHOSPHORYLATION
HALLMARK_PI3K_AKT_MTOR_SIGNALING
high risk<----------->low risk
KEGG_JAK_STAT_SIGNALING_PATHWAY
KEGG_OXIDATIVE_PHOSPHORYLATION
KEGG_WNT_SIGNALING_PATHWAY
high risk<----------->low risk

Figure S8

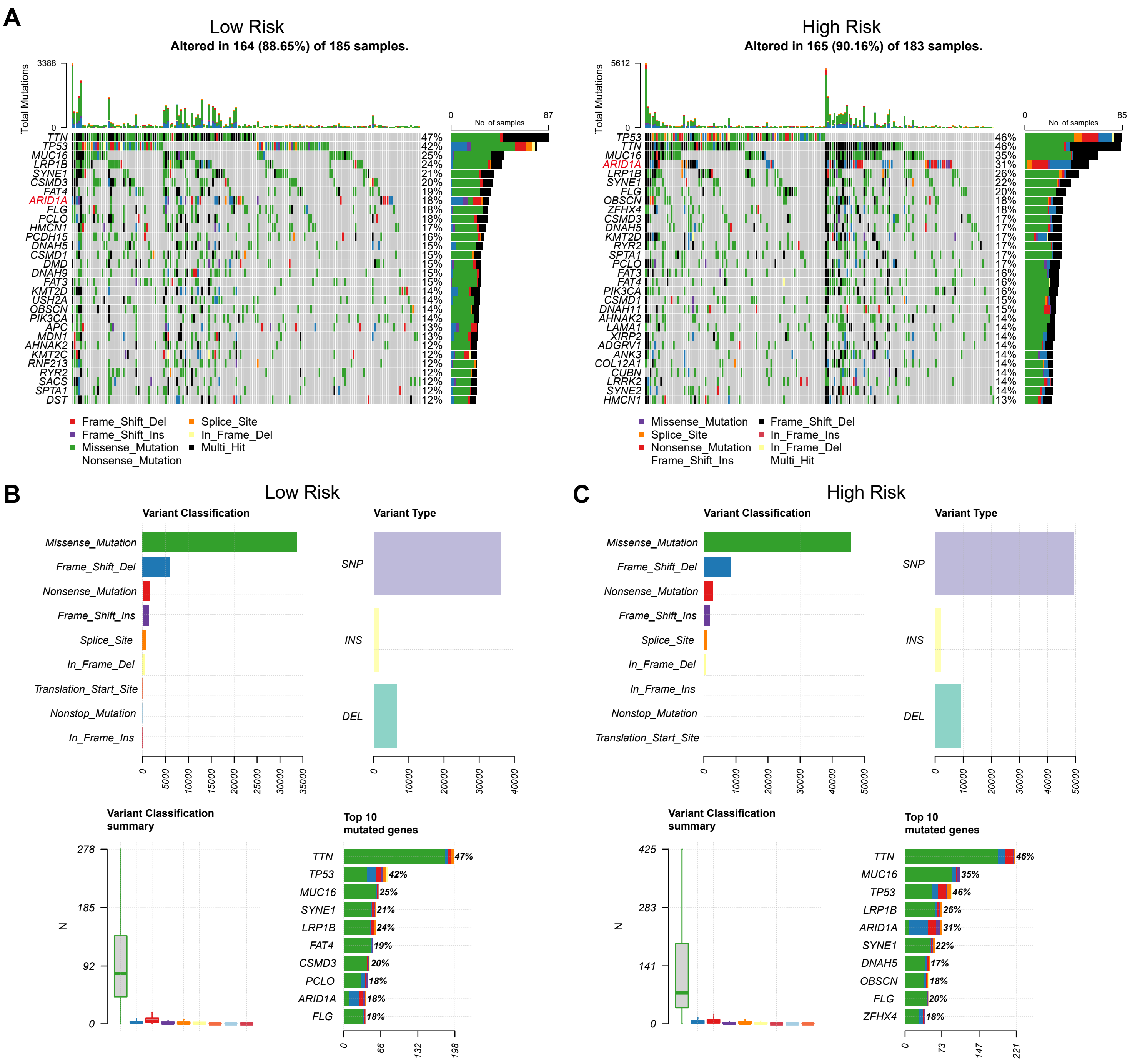
A
Low Risk
Altered in 164 (88.65%) of 185 samples.
Total Mutations
3388
0
No. of samples
87
TTN 47%
TP53 42%
MUC16 25%
LRP1B 24%
SYNE1 21%
CSMD3 20%
FAT4 19%
ARID1A 18%
FLG 18%
PCLO 18%
HMCN1 17%
PCDH15 16%
DNAH5 15%
CSMD1 15%
DMD 15%
DNAH9 15%
FAT3 15%
KMT2D 14%
USH2A 14%
OBSCN 14%
PIK3CA 14%
APC 13%
MDN1 13%
AHNAK2 12%
KMT2C 12%
RNF213 12%
RYR2 12%
SACS 12%
SPTA1 12%
DST 12%
Frame_Shift_Del
Frame_Shift_Ins
Missense_Mutation
Nonsense_Mutation
Splice_Site
In_Frame_Del
Multi_Hit
High Risk
Altered in 165 (90.16%) of 183 samples.
Total Mutations
5612
0
No. of samples
85
TP53 46%
TTN 46%
MUC16 35%
ARID1A 31%
LRP1B 26%
SYNE1 22%
FLG 20%
OBSCN 18%
ZFHX4 18%
CSMD3 17%
DNAH5 17%
KMT2D 17%
RYR2 17%
SPTA1 17%
PCLO 17%
FAT3 16%
FAT4 16%
PIK3CA 16%
CSMD1 15%
DNAH11 15%
AHNAK2 14%
LAMA1 14%
XIRP2 14%
ADGRV1 14%
ANK3 14%
COL12A1 14%
CUBN 14%
LRRK2 14%
SYNE2 14%
HMCN1 13%
Missense_Mutation
Splice_Site
Nonsense_Mutation
Frame_Shift_Ins
Frame_Shift_Del
In_Frame_Ins
In_Frame_Del
Multi_Hit
B
Low Risk
Variant Classification
Missense_Mutation
Frame_Shift_Del
Nonsense_Mutation
Frame_Shift_Ins
Splice_Site
In_Frame_Del
Translation_Start_Site
Nonstop_Mutation
In_Frame_Ins
0 5000 10000 15000 20000 25000 30000 35000
Variant Type
SNP
INS
DEL
0 10000 20000 30000 40000
Variant Classification summary
N
278
185
92
0
Top 10 mutated genes
TTN 47%
TP53 42%
MUC16 25%
SYNE1 21%
LRP1B 24%
FAT4 19%
CSMD3 20%
PCLO 18%
ARID1A 18%
FLG 18%
0 66 132 198
C
High Risk
Variant Classification
Missense_Mutation
Frame_Shift_Del
Nonsense_Mutation
Frame_Shift_Ins
Splice_Site
In_Frame_Del
In_Frame_Ins
Nonstop_Mutation
Translation_Start_Site
0 10000 20000 30000 40000 50000
Variant Type
SNP
INS
DEL
0 10000 20000 30000 40000 50000
Variant Classification summary
N
425
283
141
0
Top 10 mutated genes
TTN 46%
MUC16 35%
TP53 46%
LRP1B 26%
ARID1A 31%
SYNE1 22%
DNAH5 17%
OBSCN 18%
FLG 20%
ZFHX4 18%
0 73 147 221

Figure S9

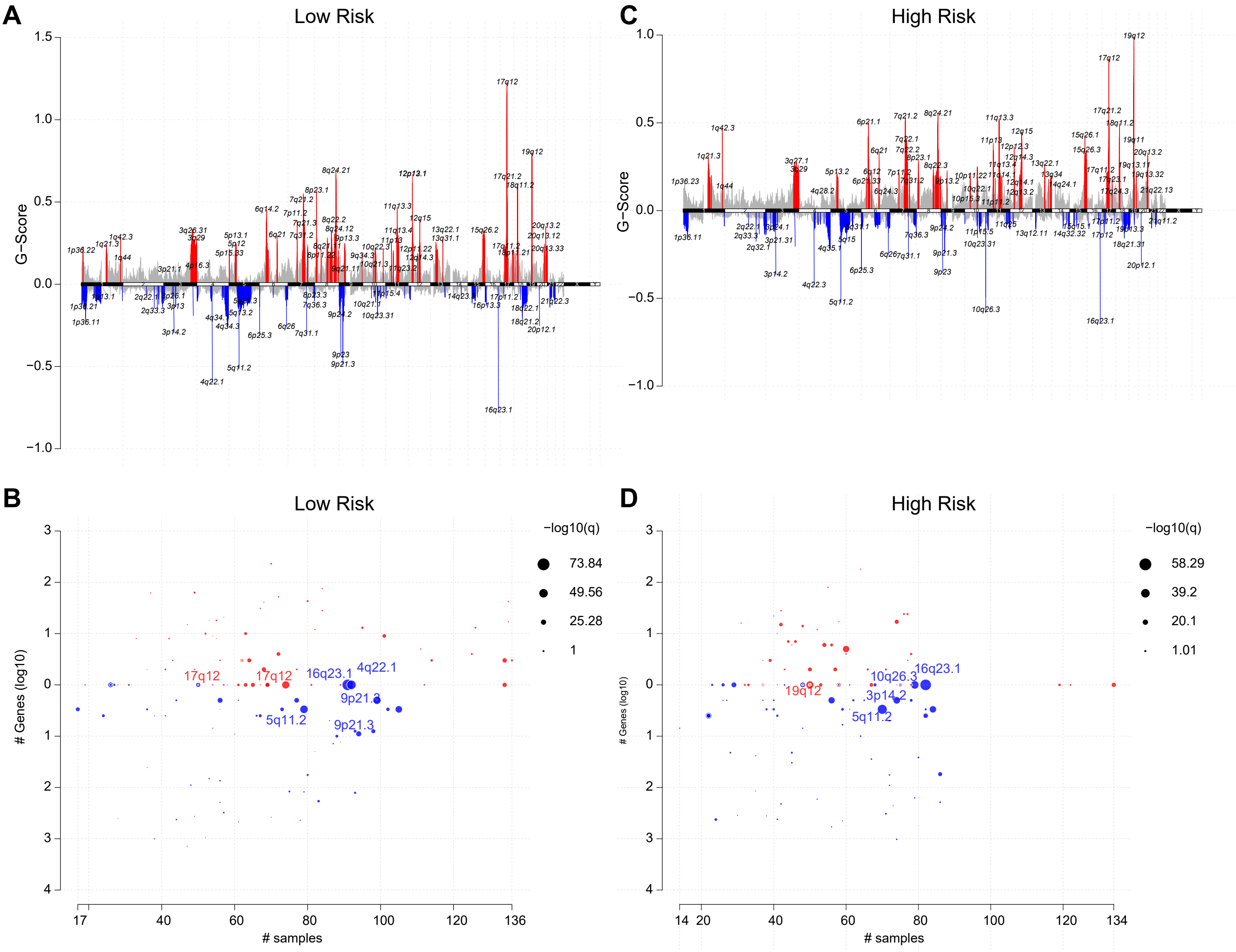

A
Low Risk
G−Score
C
High Risk
G−Score
B
Low Risk
Genes (log10)
samples
−log10(q)
73.84
49.56
25.28
1
17q12
16q23.1
4q22.1
9p21.3
5q11.2
D
High Risk
Genes (log10)
samples
−log10(q)
58.29
39.2
20.1
1.01
19q12
10q26.3
16q23.1
3p14.2
5q11.2

Figure S10

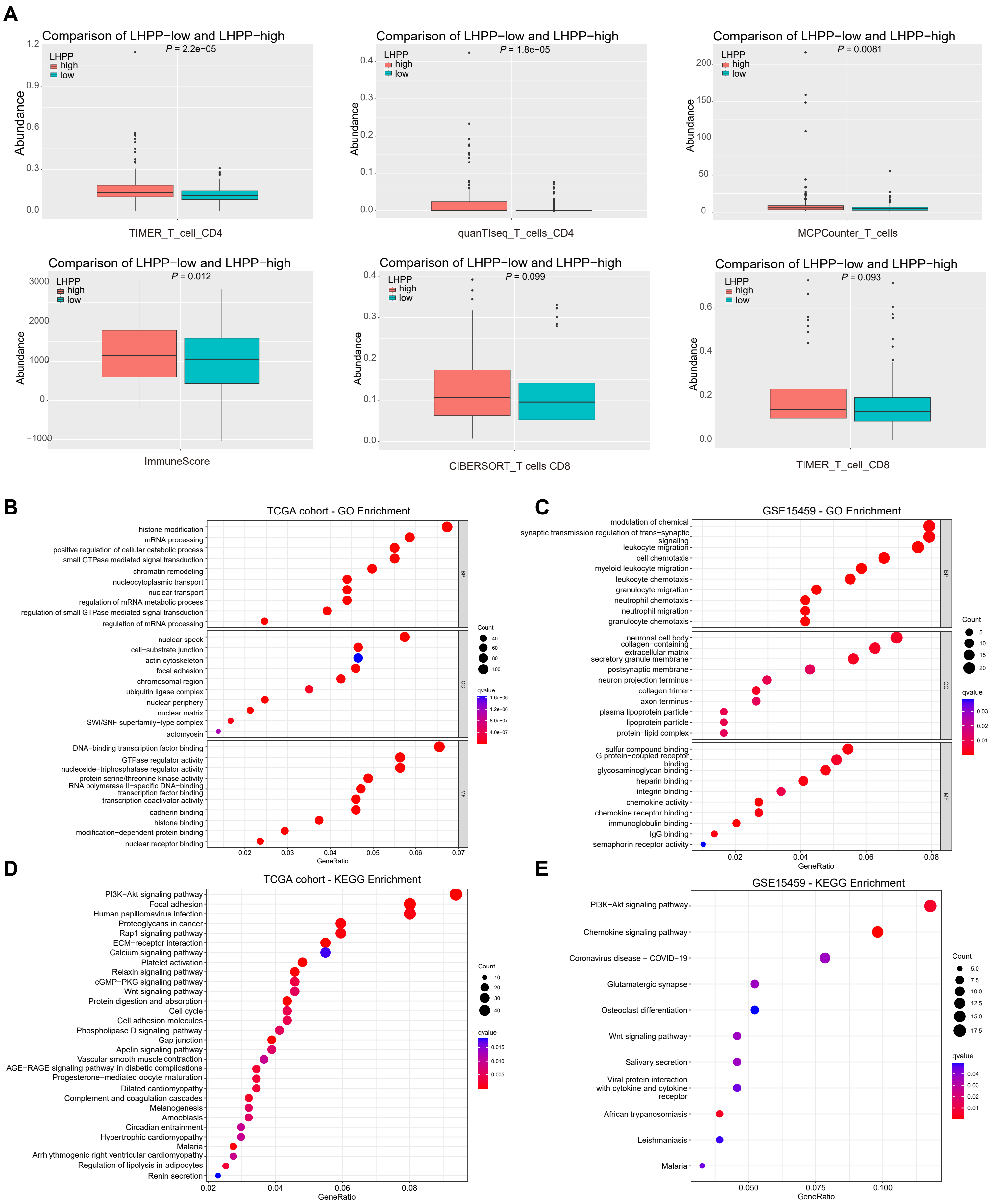

A
Comparison of LHPP−low and LHPP−high
P = 2.2e−05
LHPP
high
low
Abundance
TIMER_T_cell_CD4
Comparison of LHPP−low and LHPP−high
P = 1.8e−05
quanTIseq_T_cells_CD4
Comparison of LHPP−low and LHPP−high
P = 0.0081
MCPCounter_T_cells
Comparison of LHPP−low and LHPP−high
P = 0.012
ImmuneScore
Comparison of LHPP−low and LHPP−high
P = 0.099
CIBERSORT_T cells CD8
Comparison of LHPP−low and LHPP−high
P = 0.093
TIMER_T_cell_CD8
B
TCGA cohort - GO Enrichment
histone modification
mRNA processing
positive regulation of cellular catabolic process
small GTPase mediated signal transduction
chromatin remodeling
nucleocytoplasmic transport
nuclear transport
regulation of mRNA metabolic process
regulation of small GTPase mediated signal transduction
regulation of mRNA processing
nuclear speck
cell−substrate junction
actin cytoskeleton
focal adhesion
chromosomal region
ubiquitin ligase complex
nuclear periphery
nuclear matrix
SWI/SNF superfamily−type complex
actomyosin
DNA−binding transcription factor binding
GTPase regulator activity
nucleoside−triphosphatase regulator activity
protein serine/threonine kinase activity
RNA polymerase II−specific DNA−binding transcription factor binding
transcription coactivator activity
cadherin binding
histone binding
modification−dependent protein binding
nuclear receptor binding
BP
CC
MF
Count
qvalue
GeneRatio
C
GSE15459 - GO Enrichment
modulation of chemical synaptic transmission
regulation of trans−synaptic signaling
leukocyte migration
cell chemotaxis
myeloid leukocyte migration
leukocyte chemotaxis
granulocyte migration
neutrophil chemotaxis
neutrophil migration
granulocyte chemotaxis
neuronal cell body
collagen−containing extracellular matrix
secretory granule membrane
postsynaptic membrane
neuron projection terminus
collagen trimer
axon terminus
plasma lipoprotein particle
lipoprotein particle
protein−lipid complex
sulfur compound binding
G protein−coupled receptor binding
glycosaminoglycan binding
heparin binding
integrin binding
chemokine activity
chemokine receptor binding
immunoglobulin binding
IgG binding
semaphorin receptor activity
Count
qvalue
GeneRatio
D
TCGA cohort - KEGG Enrichment
PI3K−Akt signaling pathway
Focal adhesion
Human papillomavirus infection
Proteoglycans in cancer
Rap1 signaling pathway
ECM−receptor interaction
Calcium signaling pathway
Platelet activation
Relaxin signaling pathway
cGMP−PKG signaling pathway
Wnt signaling pathway
Protein digestion and absorption
Cell cycle
Cell adhesion molecules
Phospholipase D signaling pathway
Gap junction
Apelin signaling pathway
Vascular smooth muscle contraction
AGE−RAGE signaling pathway in diabetic complications
Progesterone−mediated oocyte maturation
Dilated cardiomyopathy
Complement and coagulation cascades
Melanogenesis
Amoebiasis
Circadian entrainment
Hypertrophic cardiomyopathy
Malaria
Arrhythmogenic right ventricular cardiomyopathy
Regulation of lipolysis in adipocytes
Renin secretion
Count
qvalue
GeneRatio
E
GSE15459 - KEGG Enrichment
PI3K−Akt signaling pathway
Chemokine signaling pathway
Coronavirus disease − COVID−19
Glutamatergic synapse
Osteoclast differentiation
Wnt signaling pathway
Salivary secretion
Viral protein interaction with cytokine and cytokine receptor
African trypanosomiasis
Leishmaniasis
Malaria
Count
qvalue
GeneRatio

Figure S11

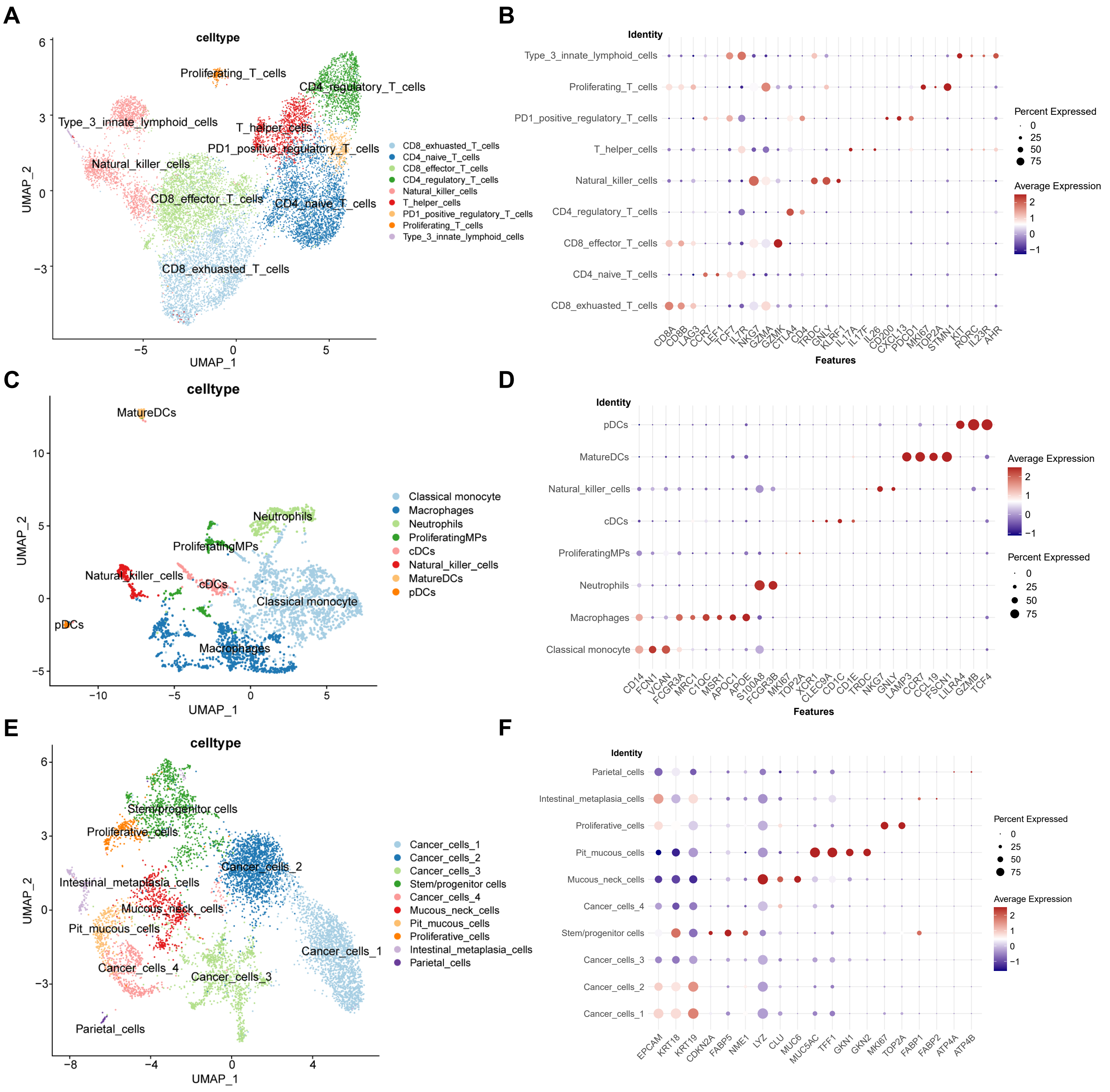
A
celltype
Proliferating_T_cells
CD4_regulatory_T_cells
Type_3_innate_lymphoid_cells
T_helper_cells
PD1_positive_regulatory_T_cells
Natural_killer_cells
CD8_effector_T_cells
CD4_naive_T_cells
CD8_exhuasted_T_cells
UMAP_1
UMAP_2
CD8_exhuasted_T_cells
CD4_naive_T_cells
CD8_effector_T_cells
CD4_regulatory_T_cells
Natural_killer_cells
T_helper_cells
PD1_positive_regulatory_T_cells
Proliferating_T_cells
Type_3_innate_lymphoid_cells
B
Identity
Features
CD8A CD8B LAG3 CCR7 LEF1 TCF7 IL7R NKG7 GZMA GZMK CTLA4 CD4 TRDC GNLY KLRF1 IL17A IL17F IL26 CD200 CXCL13 PDCD1 MKI67 TOP2A STMN1 KIT RORC IL23R AHR
Percent Expressed
Average Expression
C
celltype
MatureDCs
Neutrophils
ProliferatingMPs
Natural_killer_cells
cDCs
Classical monocyte
pDCs
Macrophages
UMAP_1
UMAP_2
Classical monocyte
Macrophages
Neutrophils
ProliferatingMPs
cDCs
Natural_killer_cells
MatureDCs
pDCs
D
Identity
Features
CD14 FCN1 VCAN FCGR3A MRC1 C1QC MSR1 APOC1 APOE S100A8 FCGR3B MKI67 TOP2A XCR1 CLEC9A CD1C CD1E TRDC NKG7 GNLY LAMP3 CCR7 CCL19 FSCN1 LILRA4 GZMB TCF4
Average Expression
Percent Expressed
E
celltype
Stem/progenitor cells
Proliferative_cells
Cancer_cells_2
Intestinal_metaplasia_cells
Mucous_neck_cells
Pit_mucous_cells
Cancer_cells_1
Cancer_cells_4
Cancer_cells_3
Parietal_cells
UMAP_1
UMAP_2
Cancer_cells_1
Cancer_cells_2
Cancer_cells_3
Stem/progenitor cells
Cancer_cells_4
Mucous_neck_cells
Pit_mucous_cells
Proliferative_cells
Intestinal_metaplasia_cells
Parietal_cells
F
Identity
EPCAM KRT18 KRT19 CDKN2A FABP5 NME1 LYZ CLU MUC6 MUC5AC TFF1 GKN1 GKN2 MKI67 TOP2A FABP1 FABP2 ATP4A ATP4B
Percent Expressed
Average Expression

Figure S12

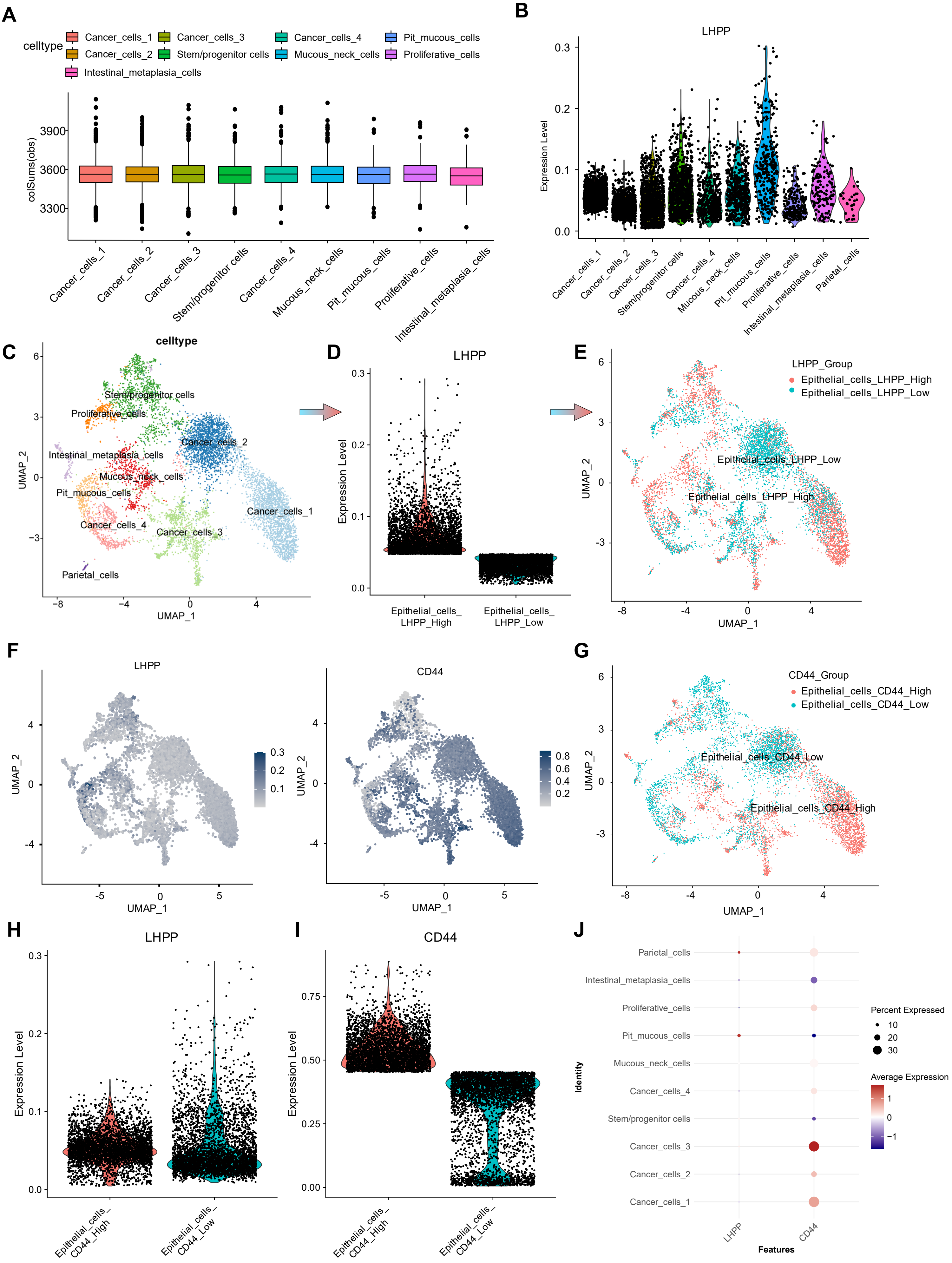
A
celltype
Cancer_cells_1
Cancer_cells_2
Cancer_cells_3
Stem/progenitor cells
Cancer_cells_4
Mucous_neck_cells
Pit_mucous_cells
Proliferative_cells
Intestinal_metaplasia_cells
colSums(obs)
B
LHPP
Expression Level
Parietal_cells
C
celltype
UMAP_1
UMAP_2
D
Epithelial_cells_LHPP_High
Epithelial_cells_LHPP_Low
E
LHPP_Group
F
CD44
G
CD44_Group
Epithelial_cells_CD44_High
Epithelial_cells_CD44_Low
H
I
J
Identity
Features
Percent Expressed
Average Expression

Figure S13

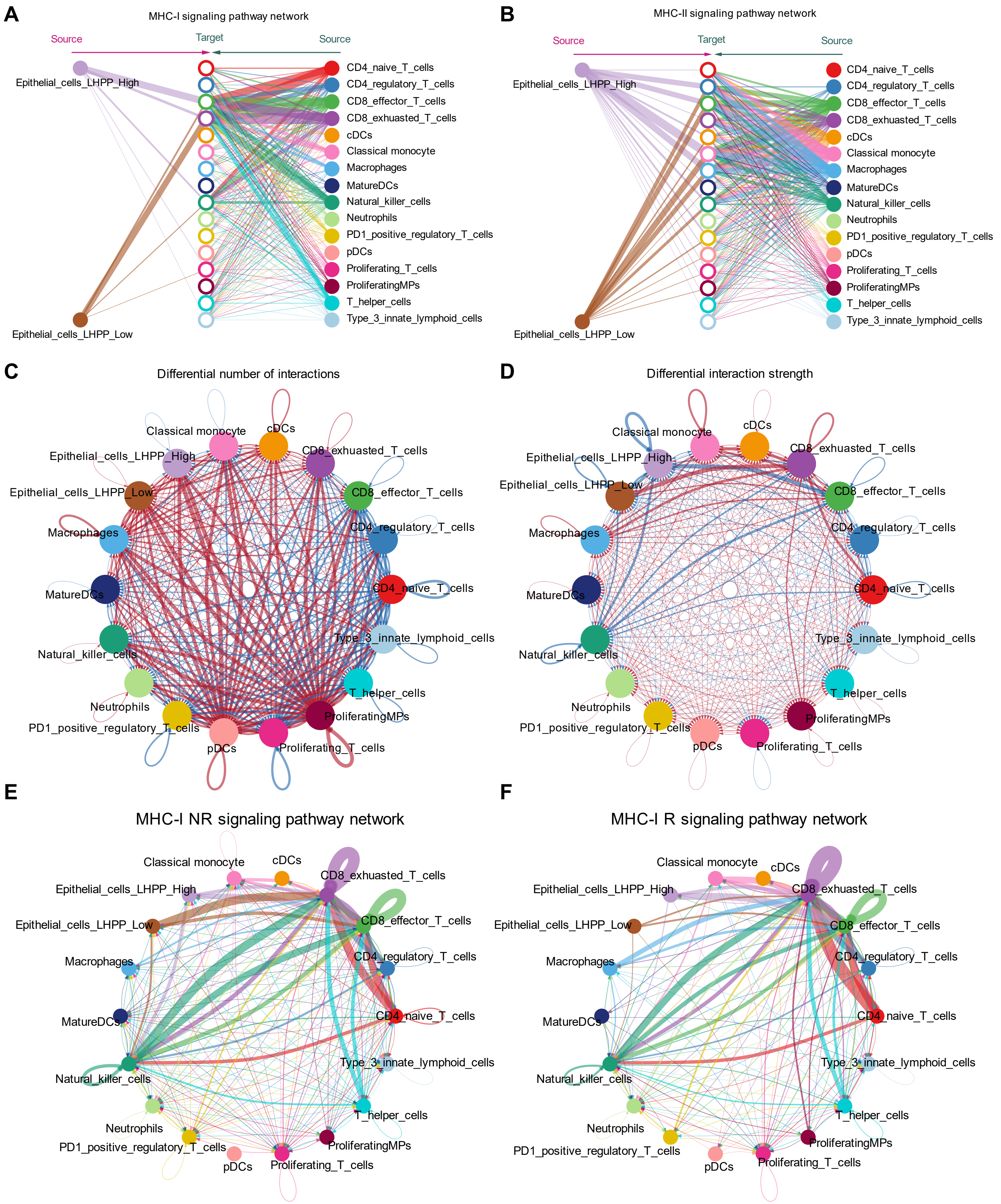
A
MHC-I signaling pathway network
Source
Target
Source
Epithelial_cells_LHPP_High
Epithelial_cells_LHPP_Low
CD4_naive_T_cells
CD4_regulatory_T_cells
CD8_effector_T_cells
CD8_exhuasted_T_cells
cDCs
Classical monocyte
Macrophages
MatureDCs
Natural_killer_cells
Neutrophils
PD1_positive_regulatory_T_cells
pDCs
Proliferating_T_cells
ProliferatingMPs
T_helper_cells
Type_3_innate_lymphoid_cells
B
MHC-II signaling pathway network
C
Differential number of interactions
D
Differential interaction strength
E
MHC-I NR signaling pathway network
F
MHC-I R signaling pathway network

Figure S14

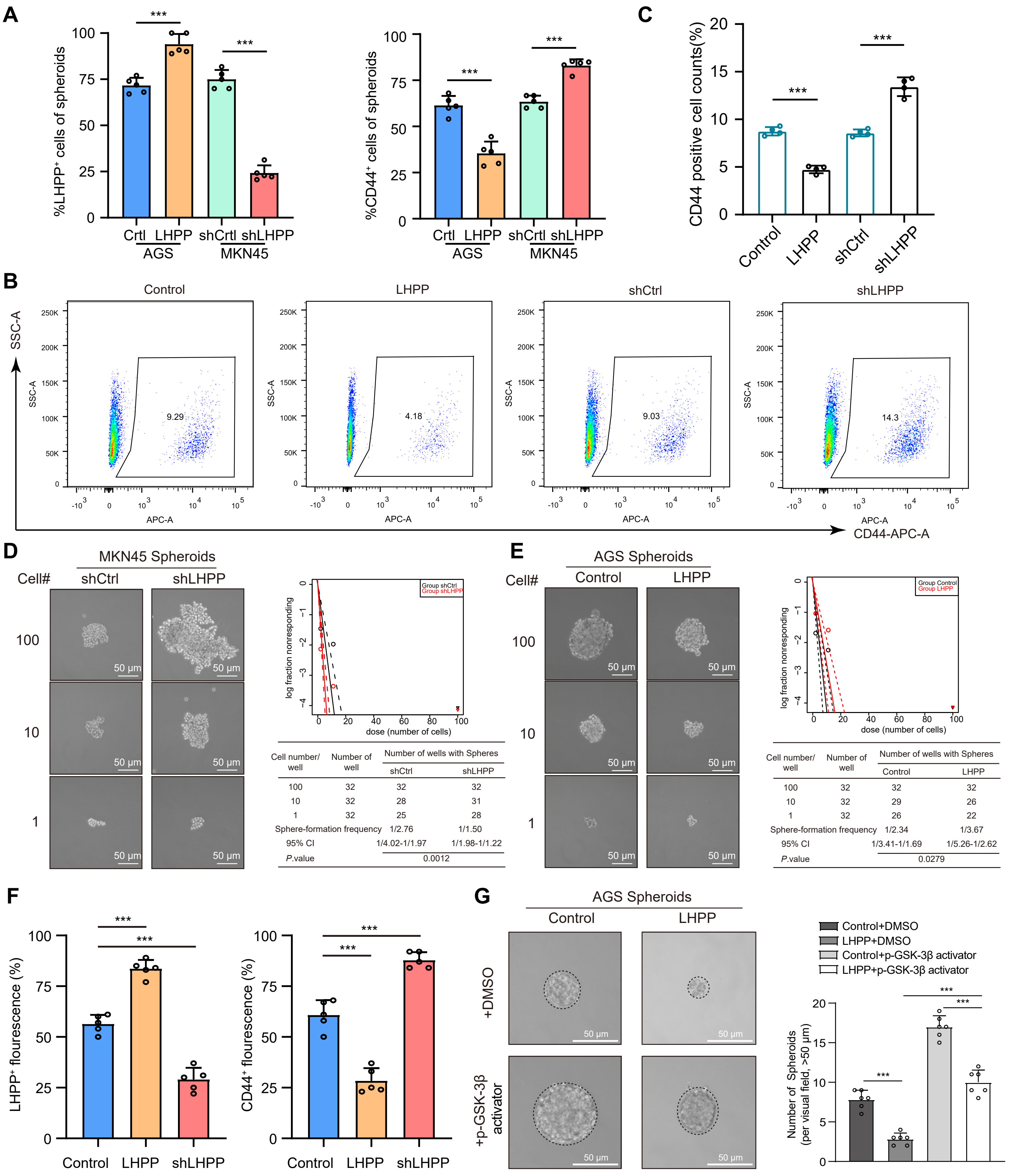

Cell number/ well	Number of well	Number of wells with Spheres	
		shCtrl	shLHPP
100	32	32	32
10	32	28	31
1	32	25	28
Sphere-formation frequency		1/2.76	1/1.50
95% CI		1/4.02-1/1.97	1/1.98-1/1.22
P.value		0.0012	

Cell number/ well	Number of well	Number of wells with Spheres	
		Control	LHPP
100	32	32	32
10	32	29	26
1	32	26	22
Sphere-formation frequency		1/2.34	1/3.67
95% CI		1/3.41-1/1.69	1/5.26-1/2.62
P.value		0.0279	

Figure S15

A

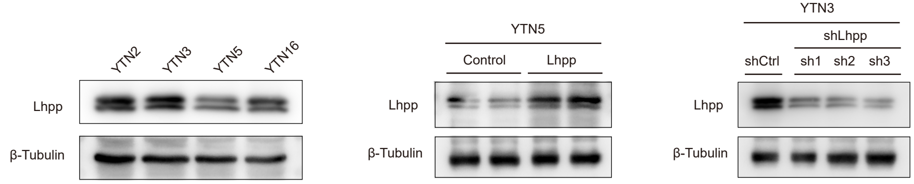

B

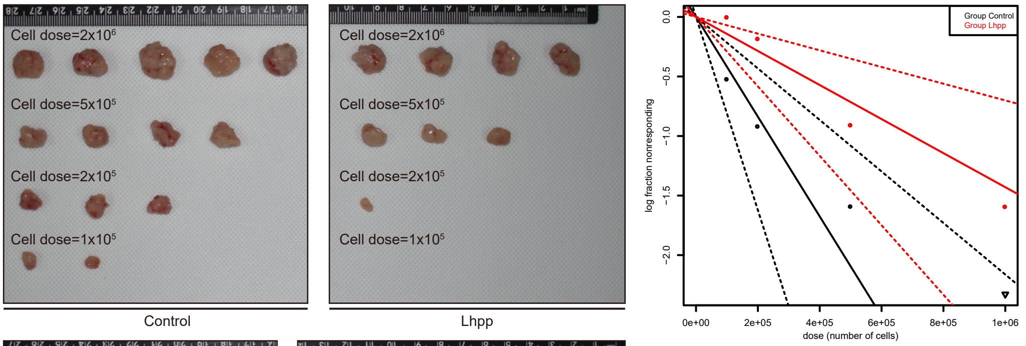

Number of cells injected (YTN5)	Number of samples tested	Number of developing tumor	
		Control	Lhpp
$2x10^6$	5	5	4
$5x10^5$	5	4	3
$2x10^5$	5	3	1
$1x10^5$	5	2	0
Sphere-formation frequency		1/238744	1/699446
95% CI		1/462354-1/123280	1/1426422-1/342973
P.value		0.0279	

C

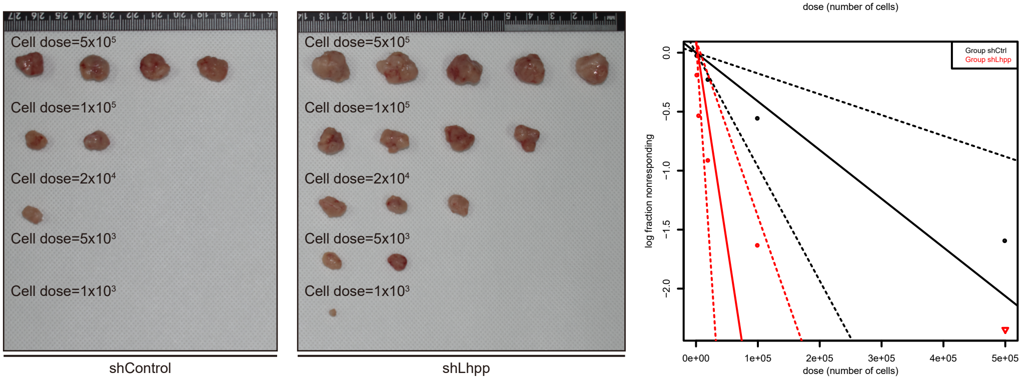

Number of cells injected (YTN3)	Number of samples tested	Number of developing tumor	
		shCtrl	shLhpp
$5x10^5$	5	4	5
$1x10^5$	5	2	4
$2x10^4$	5	1	3
$5x10^3$	5	0	2
$1x10^3$	5	0	1
Sphere-formation frequency		1/242326	1/30098
95% CI		1/567570-1/103462	1/69475-1/13040
P.value		0.000159	

D E

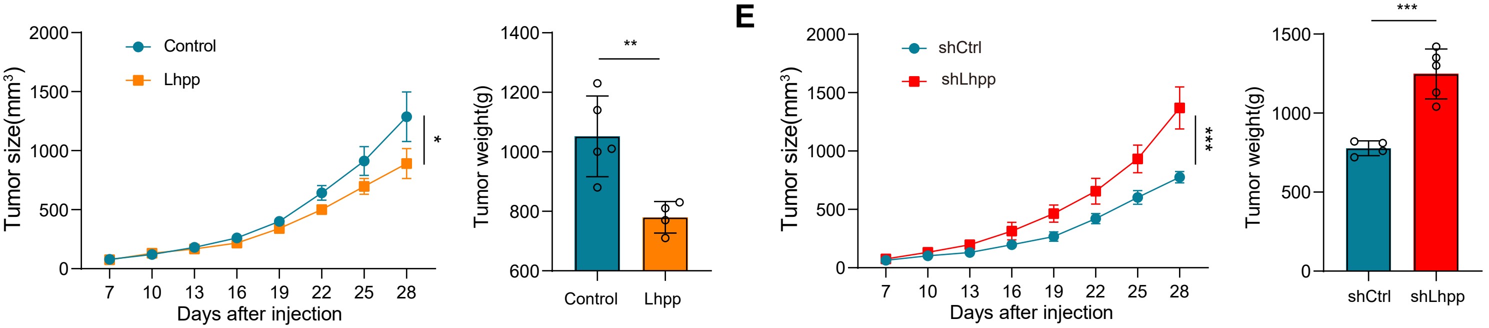

Figure S16

A

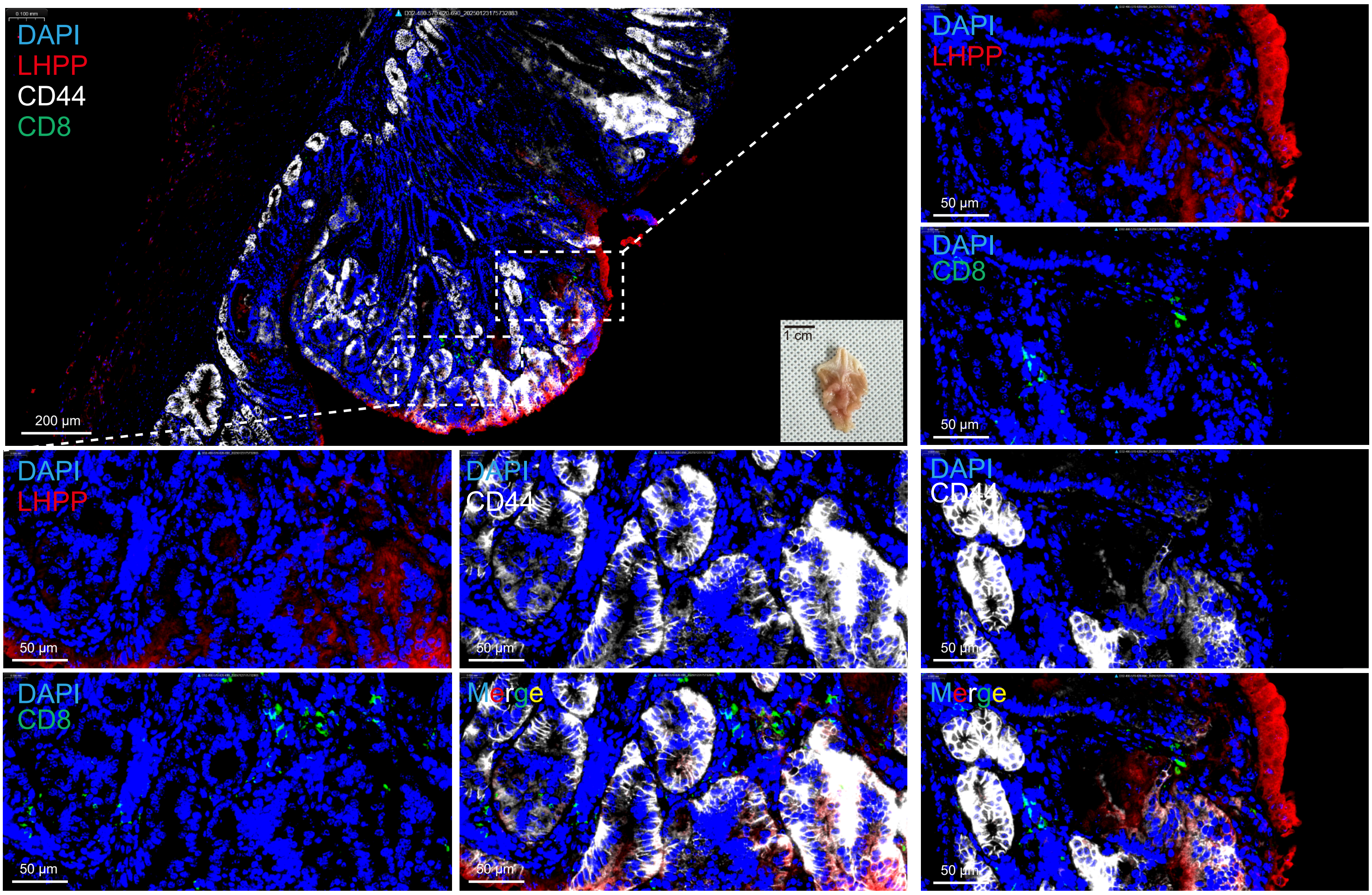

B

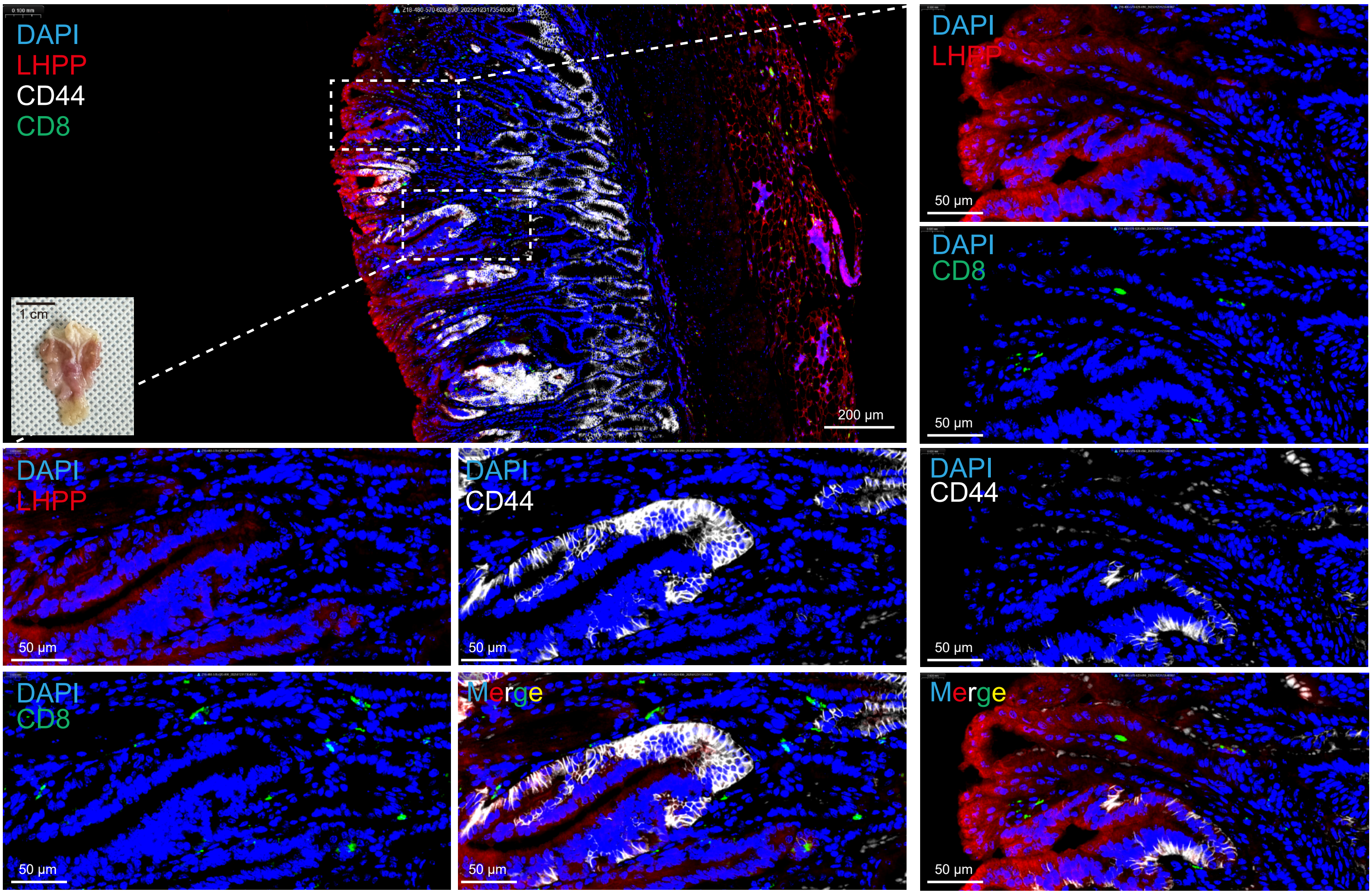

Figure S17

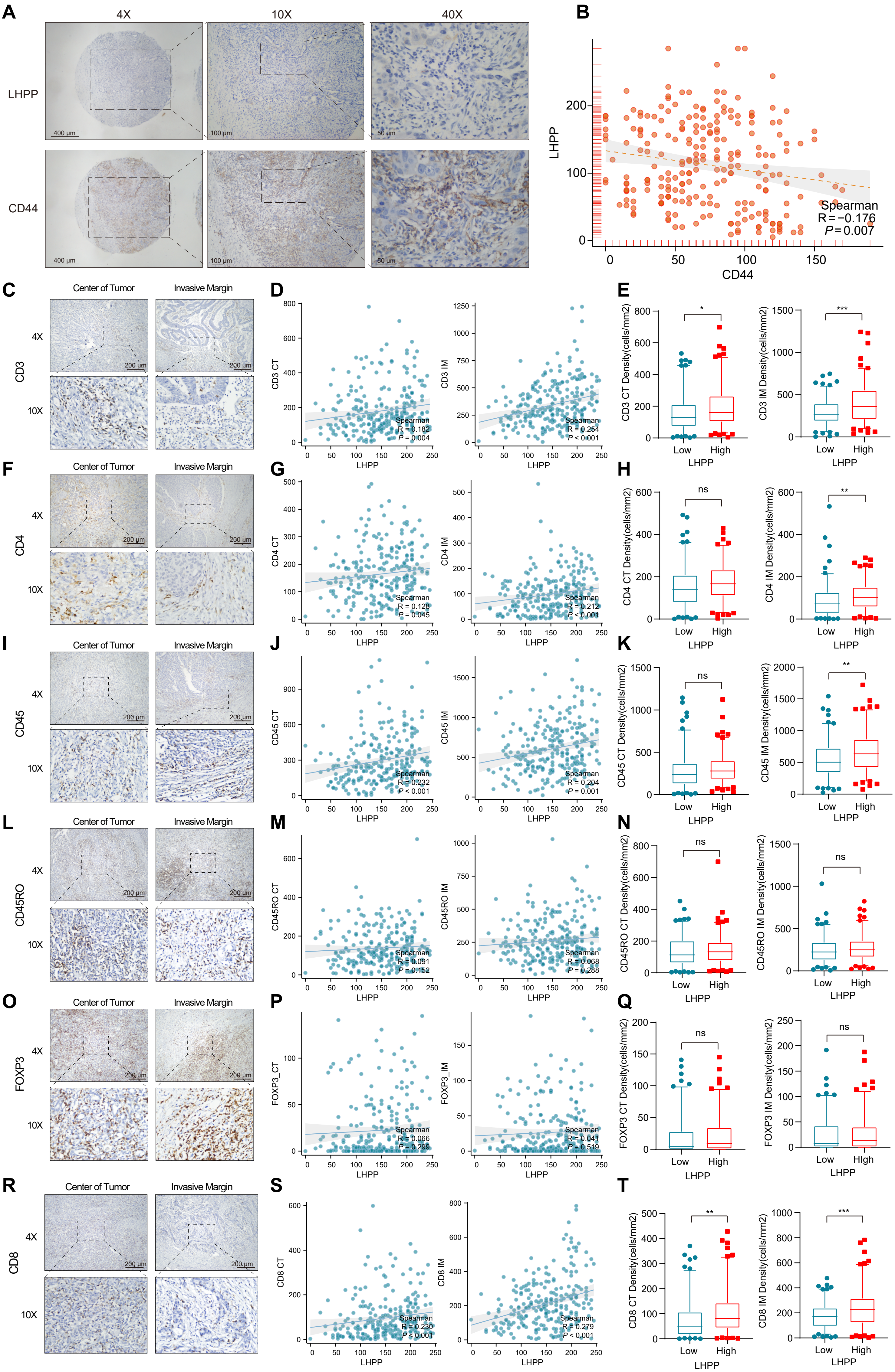
A
4X
10X
40X
LHPP
CD44
400 μm
100 μm
50 μm
B
LHPP
CD44
Spearman
R = −0.176
P = 0.007
C
Center of Tumor
Invasive Margin
CD3
D
CD3 CT
CD3 IM
LHPP
Spearman
R = 0.182
P = 0.004
R = 0.264
P < 0.001
E
CD3 CT Density(cells/mm2)
CD3 IM Density(cells/mm2)
Low
High
F
CD4
G
CD4 CT
CD4 IM
R = 0.128
P = 0.045
R = 0.212
P < 0.001
H
CD4 CT Density(cells/mm2)
CD4 IM Density(cells/mm2)
ns
I
CD45
J
CD45 CT
CD45 IM
R = 0.232
P < 0.001
R = 0.204
P = 0.001
K
CD45 CT Density(cells/mm2)
CD45 IM Density(cells/mm2)
L
CD45RO
M
CD45RO CT
CD45RO IM
R = 0.091
P = 0.152
R = 0.068
P = 0.288
N
CD45RO CT Density(cells/mm2)
CD45RO IM Density(cells/mm2)
O
FOXP3
P
FOXP3_CT
FOXP3_IM
R = 0.066
P = 0.299
R = 0.041
P = 0.519
Q
FOXP3 CT Density(cells/mm2)
FOXP3 IM Density(cells/mm2)
R
CD8
S
CD8 CT
CD8 IM
R = 0.230
P < 0.001
R = 0.279
P < 0.001
T
CD8 CT Density(cells/mm2)
CD8 IM Density(cells/mm2)

Figure S18

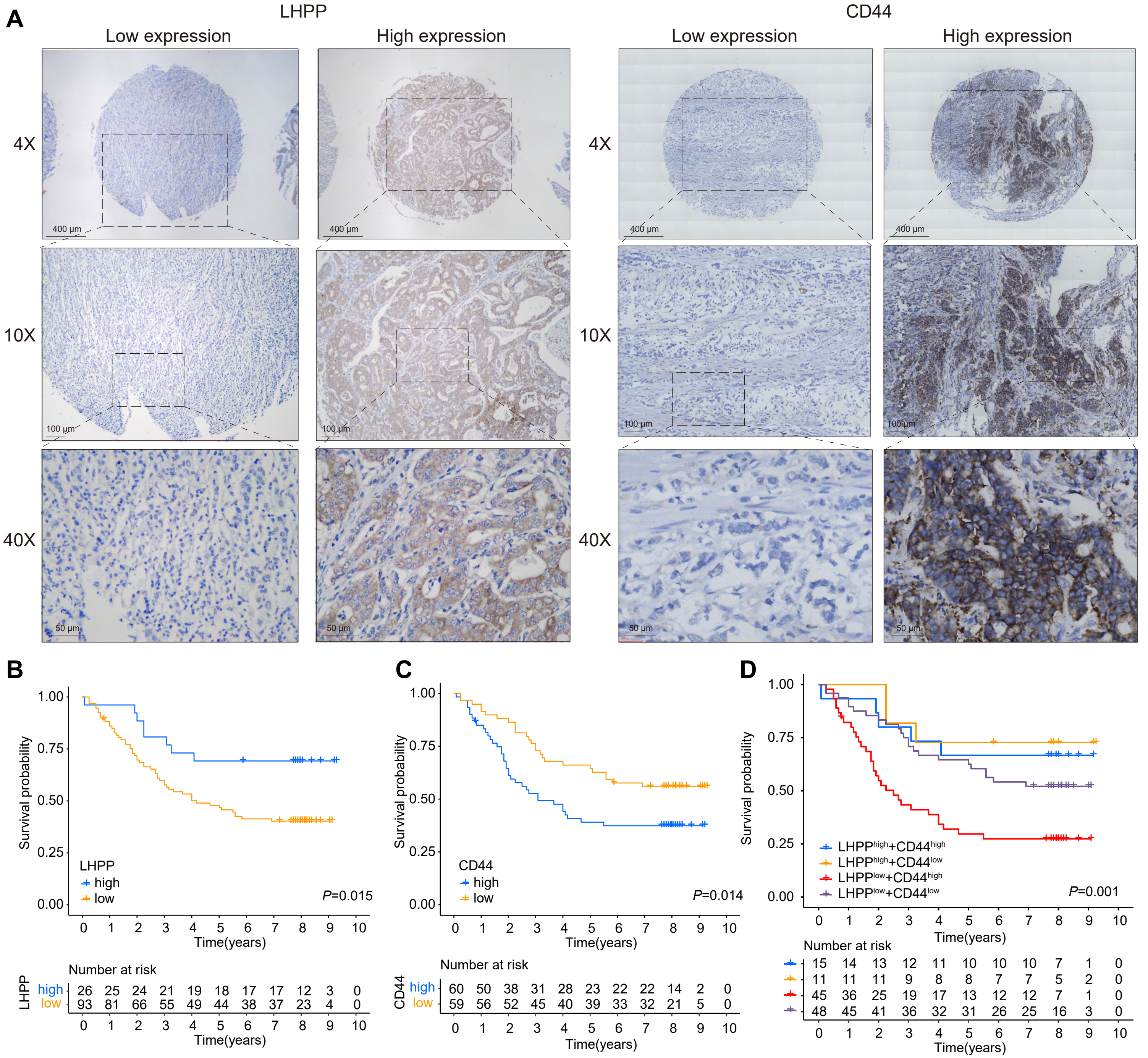
A
LHPP
CD44
Low expression
High expression
Low expression
High expression
4X
10X
40X
400 μm
100 μm
50 μm
B
Survival probability
1.00
0.75
0.50
0.25
0.00
LHPP
high
low
P=0.015
Time(years)
Number at risk
LHPP
high 26 25 24 21 19 18 17 17 12 3 0
low 93 81 66 55 49 44 38 37 23 4 0
0 1 2 3 4 5 6 7 8 9 10
C
CD44
high
low
P=0.014
CD44
high 60 50 38 31 28 23 22 22 14 2 0
low 59 56 52 45 40 39 33 32 21 5 0
D
LHPPhigh+CD44high
LHPPhigh+CD44low
LHPPlow+CD44high
LHPPlow+CD44low
P=0.001
15 14 13 12 11 10 10 10 7 1 0
11 11 11 9 8 8 7 7 5 2 0
45 36 25 19 17 13 12 12 7 1 0
48 45 41 36 32 31 26 25 16 3 0

Table S1. Relationship between Risk and baseline characteristics of patients in the TCGA cohort.

Variables	Total	Risk			
		$Risk^{low}$	$Risk^{high}$	$\chi 2$	*P*
**Gender**				0.001	0.975
Male	234	121	113		
Female	130	67	63		
**Age at surgery (years)**				0.243	0.622
<65	152	80	72		
≥65	208	104	104		
Missing	4	4	0		
**TNM stage**				5.348	0.148
I	50	30	20		
II	109	61	48		
III	148	79	69		
IV	38	14	24		
Missing	19	4	15		

Table S2. Relationship between Risk and baseline characteristics of patients in the GSE15459 cohort.

**Variables**	**Total**	**Risk**			
		**Risk^{low}**	**Risk^{high}**	$\chi 2$	***P***
**Gender**				0.151	0.698
Male	125	25	100		
Female	67	15	52		
**Age at surgery (years)**				0.289	0.591
<65	84	16	68		
≥65	108	24	84		
**TNM stage**				4.668	0.198
I	31	9	22		
II	29	9	20		
III	72	11	61		
IV	60	11	49		

Table S3. List of primers used for genotyping.

Name	Sequence
Mist1-CreERT2 #1	AGCGCCTAGGTGTCCACTAA
Mist1-CreERT2 #2	CATTGCTGTCACTTGGTCGT
Mist1-CreERT2 #3	GACTGGGGTCTGTCAGGTGT
Apc flox #1	CAAAGCACACTTTGGCAGACT
Apc flox #2	GCTTATTTATACCCTTGGCAATC
p53 flox #1	GGTTAAACCCAGCTTGACCA
p53 flox #2	GGAGGCAGAGACAGTTGGAG
Rosa26Tdtomato #1	CTGTTCCTGTACGGCATGG
Rosa26Tdtomato #2	GGCATTAAAGCAGCGTATCC
