# Supplementary material for: Targeting LHPP in neoadjuvant chemotherapy resistance of gastric cancer: insights from single-cell and multi-omics data on tumor immune microenvironment and stemness characteristics
Source: Cell Death Dis. 2025 Apr 16;16(1):306. doi: 10.1038/s41419-025-07614-z (PMC12003742; doi:10.1038/s41419-025-07614-z)
Supplement: Supplementary file 2 — Supplementary Figure Legends [file 41419_2025_7614_MOESM2_ESM.pdf]

Figure S1 (A) Unsupervised clustering consensus matrices for k = 1 to k = 8, used to identify distinct stemness subgroups within the dataset. The consensus matrix legend (left) shows the degree of clustering stability, with darker blue indicating higher consensus among samples within the same cluster. **(B)** Box plot comparing mRNAsi scores between the High and Low stemness subgroups. Statistical significance was determined using a p-value. **(C)** Box plots displaying immune cell infiltration levels across different immune cell types between the High and Low stemness subgroups. Immune infiltration was assessed for various cell types. Student's t-test was used to compare the statistical difference between the two groups. *, $P < 0.05$; **, $P < 0.01$; ***, $P < 0.001$.

Figure S2 (A) Unsupervised clustering consensus matrices for k = 1 to k = 8, used to identify distinct immune subgroups (high and low) within the dataset. The consensus matrix legend (left) illustrates the degree of clustering stability, with darker blue indicating higher consensus among samples within the same cluster. **(B)** Box plots comparing stemness scores across various stemness-related pathways and gene sets between high and low immune subgroups. Student's t-test was used to compare the statistical difference between the two groups. *, $P < 0.05$; **, $P < 0.01$; ***, $P < 0.001$.

Figure S3 Integrative analysis of stemness and immune characteristics in the transcriptome of gastric cancer patients in the GSE15459 cohort. (A) The integrated heatmap illustrates the stemness pathway scores for each patient across the two stemness phenotypes. **(B)** The integrated heatmap displays the immune cell

infiltration profiles for each patient across the two immune phenotypes. **(C)** Student's t-test was used to evaluate the differences in stemness pathway scores between the two stemness subgroups. The upper and lower edges of the box represent the interquartile range, the line within the box denotes the median, and the dots indicate outliers. **(D)** Student's t-test was used to assess differences in immune cell infiltration between the two stemness subgroups. The upper and lower edges of the box represent the interquartile range, the line within the box indicates the median, and the dots denote outliers. **(E)** Student's t-test was used to assess differences in immune cell infiltration between the two immune subgroups. The upper and lower edges of the box represent the interquartile range, the line within the box indicates the median, and the dots denote outliers. **(F)** Student's t-test was used to evaluate the differences in stemness pathway scores between the two immune subgroups. The upper and lower edges of the box represent the interquartile range, the line within the box denotes the median, and the dots indicate outliers. **(G)** GO enrichment analysis shows the pathway enrichment of differentially expressed genes in the High Stemness group compared to the Low Stemness group. **(H)** GO enrichment analysis shows the pathway enrichment of differentially expressed genes in the High Immune Subtype compared to the Low Immune Subtype.

Figure S4 Integrative analysis of stemness and immune characteristics in the transcriptome of gastric cancer patients in the sequencing data from our center. (A) The integrated heatmap illustrates the stemness pathway scores for each

patient across the two stemness phenotypes. **(B)** The integrated heatmap displays the immune cell infiltration profiles for each patient across the two immune phenotypes. **(C)** Student's t-test was used to evaluate the differences in stemness pathway scores between the two stemness subgroups. The upper and lower edges of the box represent the interquartile range, the line within the box denotes the median, and the dots indicate outliers. **(D)** Student's t-test was used to assess differences in immune cell infiltration between the two stemness subgroups. The upper and lower edges of the box represent the interquartile range, the line within the box indicates the median, and the dots denote outliers. **(E)** Student's t-test was used to assess differences in immune cell infiltration between the two immune subgroups. The upper and lower edges of the box represent the interquartile range, the line within the box indicates the median, and the dots denote outliers. **(F)** Student's t-test was used to evaluate the differences in stemness pathway scores between the two immune subgroups. The upper and lower edges of the box represent the interquartile range, the line within the box denotes the median, and the dots indicate outliers. **(G)** GO enrichment analysis shows the pathway enrichment of differentially expressed genes in the High Stemness group compared to the Low Stemness group. **(H)** GO enrichment analysis shows the pathway enrichment of differentially expressed genes in the High Immune Subtype compared to the Low Immune Subtype.

Figure S5 (A) Sankey diagram illustrating the relationships among various clinical and pathological characteristics, including stemness, immune subtype, survival status,

pathologic T stage, pathologic N stage, pathologic M stage, overall pathological stage, and neoplasm histologic grade in the TCGA cohort. **(B)** Representative hematoxylin and eosin (HE) staining images showing Tumor Regression Grade (TRG) classifications (TRG 1a, TRG 1b, TRG 2, and TRG 3) from the neoadjuvant therapy cohort at our centre. Images are displayed at 4X and 40X magnifications, with scale bars indicating 200 μm in the 4X images.

Figure S6 (A) Determination of the soft-thresholding power for network construction in weighted gene co-expression network analysis (WGCNA). The left plot shows the scale independence as a function of the soft-thresholding power, with a chosen power where the model achieves an approximate scale-free topology ($R^2 > 0.85$). The right plot illustrates mean connectivity as a function of the soft-thresholding power. **(B)** Gene dendrogram generated by WGCNA, displaying the hierarchical clustering of genes based on topological overlap, with assigned module colors below indicating different gene modules identified by dynamic tree cutting and merged dynamic tree cutting. **(C)** Scatter plot showing the correlation between module membership and gene significance within the brown module, with the correlation coefficient and p-value provided. **(D)** KEGG pathway enrichment analysis for the Brown module. The bar plot displays the enriched pathways within the Brown module, with the x-axis representing the count of genes involved in each pathway and the color gradient indicating the q-value significance level. Key pathways include the PD-L1 expression and PD-1 checkpoint pathway in cancer, focal adhesion, Wnt signaling pathway, ECM-receptor interaction, and

T cell receptor signaling pathway, among others. Pathways with lower q-values are represented in red, indicating higher significance.

Figure S7 (A) TIDE response prediction for the TCGA cohort. The bar plot shows TIDE scores for each patient, classified as responders (red) or non-responders (blue) based on a threshold value of -0.22. **(B)** TIDE response prediction for the FJMUUH cohort. Similar to panel A, patients are classified into responders (red) and non-responders (blue) using a threshold value of -0.22. **(C)** Gene Set Enrichment Analysis (GSEA) plots for hallmark and KEGG pathways in the TCGA cohort. Key pathways associated with high and low risk groups are shown, with notable enrichment in pathways such as Hypoxia, IL2-STAT5 signaling, PI3K-AKT-MTOR signaling, and Wnt signaling pathway. **(D)** GSEA plots for hallmark and KEGG pathways in the GSE15459 cohort. Pathways enriched in high and low risk groups include Oxidative Phosphorylation, PI3K-AKT-MTOR signaling, JAK-STAT signaling pathway, and Wnt signaling pathway.

Figure S8 (A) Oncoplot showing the landscape of somatic mutations in the Low Risk group (left) and High Risk group (right). The plot displays the frequency and types of mutations across top mutated genes in each group. Mutation types are color-coded, including missense mutations, nonsense mutations, frame shift deletions/insertions, splice site mutations, in-frame deletions/insertions, and multi-hit mutations. **(B)** Variant classification and type distribution in the Low Risk group. The left panel shows the distribution of variant classifications, with the most frequent mutation type being

missense mutations. The right panel illustrates the variant types, highlighting the predominance of SNPs (single nucleotide polymorphisms) and deletions (DEL). The bottom panels summarize the variant classification and top 10 mutated genes in the Low Risk group. **(C)** Variant classification and type distribution in the High Risk group, structured similarly to panel B. The classification distribution is dominated by missense mutations, with SNPs and insertions (INS) being the most common variant types. The bottom panels provide a summary of variant classifications and the top 10 mutated genes in the High Risk group.

Figure S9 (A) GISTIC analysis of copy number alterations in the Low Risk group, with chromosomal regions displaying amplification (red) and deletion (blue) frequencies. The G-score reflects the magnitude of alterations across the genome. **(B)** Bubble plot showing the frequency and significance of focal amplification (red) and deletion (blue) events in the Low Risk group. The size of the bubbles represents the significance level (-log10(q)), with chromosomal regions of interest labeled. **(C)** GISTIC analysis of copy number alterations in the High Risk group, indicating amplification (red) and deletion (blue) frequencies across the genome. **(D)** Bubble plot for the High Risk group, illustrating the frequency and significance of focal amplification (red) and deletion (blue) events. Bubble size corresponds to significance levels (-log10(q)), with notable chromosomal regions highlighted.

Figure S10 (A) Box plots comparing the abundance of immune cell types between

LHPP-low and LHPP-high expression groups across various immune cell quantification tools. Student's t-test was used to compare the statistical difference between the two groups. **(B)** GO enrichment analysis for the TCGA cohort. The bubble plot shows enriched GO terms across three categories: Biological Process, Cellular Component, and Molecular Function. The x-axis represents the GeneRatio, while the bubble size reflects the number of genes, and the color indicates the q-value significance level. **(C)** GO enrichment analysis for the GSE15459 cohort, similarly displaying enriched GO terms within the categories of Biological Process, Cellular Component, and Molecular Function. **(D)** KEGG pathway enrichment analysis for the TCGA cohort. The bubble plot illustrates the top enriched pathways, with bubble size indicating the gene count, and color indicating the q-value significance. Key pathways include PI3K-Akt signaling, ECM-receptor interaction, and calcium signaling. **(E)** KEGG pathway enrichment analysis for the GSE15459 cohort. The plot shows enriched pathways such as PI3K-Akt signaling, chemokine signaling, and Wnt signaling, with bubble size corresponding to gene count and color indicating significance level (q-value). *, $P < 0.05$; **, $P < 0.01$; ***, $P < 0.001$.

Figure S11 (A) UMAP plot showing clustering of T cell subpopulations within the tumor microenvironment, including CD8+ effector T cells, CD8+ exhausted T cells, CD4+ naive T cells, CD4+ regulatory T cells, T helper cells, natural killer cells, and various other immune cell types. **(B)** Dot plot displaying the expression levels of selected marker genes across T cell subpopulations shown in panel A. Dot size represents the percentage of cells expressing each marker, while color intensity reflects average expression levels. **(C)**

UMAP plot illustrating the distribution of myeloid cell subtypes, such as classical monocytes, macrophages, neutrophils, proliferating monocyte progenitors (MPs), dendritic cells (DCs), and plasmacytoid dendritic cells (pDCs). **(D)** Dot plot showing marker gene expression for the myeloid cell subtypes presented in panel C. Dot size indicates the percentage of cells expressing each gene, and color intensity represents the average expression. **(E)** UMAP plot depicting epithelial and stem cell subpopulations within the tumor, including cancer cells, proliferative cells, stem/progenitor cells, and various specialized epithelial cells (e.g., parietal, mucous neck, and intestinal metaplasia cells). **(F)** Dot plot illustrating the expression of marker genes across epithelial and stem cell subpopulations from panel E. The size of the dots reflects the percentage of cells expressing each marker, while color intensity indicates the average expression.

Figure S12 (A) Copy Number Variation (CNV) analysis showing the distribution of CNV counts (obs) across different epithelial and cancer cell subpopulations, including cancer cells, stem/progenitor cells, mucous neck cells, proliferative cells, pit mucous cells, and intestinal metaplasia cells. **(B)** Violin plot illustrating the expression levels of LHPP across various epithelial and cancer cell subpopulations, indicating differential expression among these cell types. **(C)** UMAP plot depicting the clustering of epithelial and cancer cell subpopulations, with distinct clusters for cell types such as cancer cells, stem/progenitor cells, mucous neck cells, and others. **(D)** Violin plot comparing LHPP expression between high and low LHPP-expressing epithelial cells, categorized as LHPP^{high} epithelial cells and LHPP^{low} epithelial cells. **(E)** UMAP plot illustrating the spatial

distribution of $LHPP^{high}$ epithelial cells and $LHPP^{low}$ epithelial cell groups, providing a visual representation of LHPP expression patterns within the epithelial cell population. **(F)** UMAP plots showing the expression levels of LHPP (left) and CD44 (right) across cell populations. Color intensity represents expression levels, with darker shades indicating higher expression. **(G)** UMAP plot illustrating the distribution of epithelial cells categorized by CD44 expression levels into Epithelial_cells_CD44_High (red) and Epithelial_cells_CD44_Low (cyan). **(H)** Violin plot comparing LHPP expression levels between Epithelial_cells_CD44_High and Epithelial_cells_CD44_Low, showing distinct differences in LHPP expression based on CD44 status. **(I)** Violin plot comparing CD44 expression levels between Epithelial_cells_CD44_High and Epithelial_cells_CD44_Low groups, highlighting the differential expression patterns of CD44. **(J)** Dot plot illustrating the expression levels of LHPP and CD44 across various cell identities, including parietal cells, intestinal metaplasia cells, proliferative cells, and various cancer cell subtypes.

Figure S13 (A) MHC-I signaling pathway network showing cell-cell communication interactions between epithelial cells with high and low LHPP expression (Epithelial_cells_LHPP_High and Epithelial_cells_LHPP_Low) and various immune cell types, including CD4+ naive T cells, CD8+ effector T cells, natural killer cells, and dendritic cells. The thickness of the lines indicates the strength of the interactions. **(B)** MHC-II signaling pathway network illustrating cell-cell communication between Epithelial_cells_LHPP_High and Epithelial_cells_LHPP_Low and immune cell subtypes. As in panel A, line thickness represents the interaction strength, with notable interactions

between epithelial cells and T cell subtypes, classical monocytes, macrophages, and other immune cells. **(C)** Network illustrating the differential number of interactions between Epithelial_cells_LHPP_High, Epithelial_cells_LHPP_Low, and various immune cell types, highlighting cell-cell communication within the tumor microenvironment. Nodes represent cell types, and line thickness indicates the number of interactions. **(D)** Network showing differential interaction strength between Epithelial_cells_LHPP_High, Epithelial_cells_LHPP_Low, and immune cell populations. The thickness of lines corresponds to the relative strength of interactions, emphasizing variations in communication intensity among cell types. **(E)** MHC-I signaling pathway network in neoadjuvant-resistant (NR) samples, depicting interactions between epithelial cells with varying LHPP expression levels and immune cells. This network shows communication patterns of resistance to neoadjuvant treatment. **(F)** MHC-I signaling pathway network in neoadjuvant-responsive (R) samples, illustrating cell-cell interactions for epithelial cells with high and low LHPP expression. This network reveals communication patterns associated with sensitivity to neoadjuvant therapy

Figure S14 **(A)** Quantification of immunofluorescence staining in 3D spheroid cultures, showing the percentages of *LHPP*+ cells and *CD44*+ cells in AGS and MKN45 gastric cancer cell lines. Student's t-test was used to compare the statistical difference between the two groups. **(B)** Flow cytometry analysis of *CD44* expression in AGS cells under different conditions, showing representative dot plots for control, *LHPP* overexpression, shCtrl, and sh*LHPP* groups. **(C)** Quantification of *CD44*-positive cell

counts from the flow cytometry data in panel B, comparing the percentage of *CD44*+ cells across treatment groups. Student's t-test was used to compare the statistical difference between the two groups. **(D)** Extreme limiting dilution analysis (ELDA) of spheroid formation in MKN45 cells with *LHPP* knockdown (shLHPP) compared to control (shCtrl). Representative images of spheroids at varying cell densities (100, 10, and 1 cell per well) are shown on the left (scale bar = 50 μm). The right plot displays the proportion of wells with successful spheroid formation. **(E)** ELDA in AGS cells overexpressing *LHPP* compared to control. Images on the left illustrate spheroids at cell densities of 100, 10, and 1 cell per well (scale bar = 50 μm). The right plot shows the proportion of wells with spheroid formation. **(F)** Quantification of *LHPP*+ and *CD44*+ cell percentages in spheroid cultures. Student's t-test was used to compare the statistical difference between the two groups. **(G)** Sphere formation in AGS cells with *LHPP* overexpression and GSK-3β activator treatment compared to DMSO control. Representative spheroid images are shown on the left, and the graph on the right quantifies the proportion of wells with spheroid formation. Student's t-test was used to compare the statistical difference between the two groups. *, $P < 0.05$; **, $P < 0.01$; ***, $P < 0.001$.

Figure S15 (A) Basic protein expression of *Lhpp* in four mouse GC cell lines, YTN2, YTN3, YTN5, and YTN16 was detected by western blotting. Stable *Lhpp*-overexpressing YTN5 cells and *Lhpp*-knockdown YTN3 cells were constructed. Western blotting confirmed the changes in *Lhpp* expression. In *Lhpp*-knockdown YTN3 mouse GC cells, three shRNA target sites were designed. We selected the sh3 target, which

exhibited the most significant knockdown efficiency, for subsequent experiments involving *Lhpp*-knockdown YTN3 mouse GC cells. **(B)** Tumor formation frequency at different cell inoculation densities in the C57BL/6 mouse xenograft model with upregulated *Lhpp* expression in YTN5 cells. **(C)** Tumor formation frequency at different cell inoculation densities in the C57BL/6 mouse xenograft model with downregulated *Lhpp* expression in YTN3 cells. **(D)** Tumour weight and volume changes were recorded in xenograft models of YTN5 cells with a cell dose of 2×10^6. Student's t-test was used to compare the differences between the two groups. **(E)** Tumour weight and volume changes were recorded in xenograft models of YTN3 cells with a cell dose of 5×10^5. Student's t-test was used to compare the differences between the two groups.

Figure S16 (A, B) Representative images of gastric cancer in the *Mist-CreERT;Apc*$^{fl/fl}$*;p53*$^{fl/fl}$*;Rosa26*^{Tdtomato} conditional knockout gene mouse model. Multiplex immunofluorescence staining was used to assess the expression of *LHPP*, *CD44*, CD8, and DAPI in the model.

Figure S17 (A) Representative immunohistochemical (IHC) images showing the expression of *LHPP* (top row) and *CD44* (bottom row) in gastric cancer tissue at 4X, 10X, and 40X magnifications. The images reveal distinct expression patterns, with high *LHPP* and *CD44* staining seen in the tumor microenvironment. Scale bar = 400 μm (4X magnification). **(B)** Spearman correlation analysis between *LHPP* and *CD44* expression levels across gastric cancer samples. **(C, F, I, L, O, R)** Representative

immunohistochemical (IHC) images at 4X and 10X magnifications showing the expression of CD3, CD4, CD45, CD45RO, FOXP3, and CD8 immune markers in the center of the tumor and invasive margin of gastric cancer tissues. **(D, G, J, M, P, S)** Correlation scatter plots illustrating the relationship between *LHPP* expression and the density of CD3, CD4, CD45, CD45RO, FOXP3, and CD8 positive immune cells in both the center of the tumor (CT) and invasive margin (IM). Significant correlations are noted for CD3, CD4 and CD8 densities with *LHPP* levels. All correlation analyses were performed using Spearman correlation coefficient for statistical analysis. **(E, H, K, N, Q, T)** Box plots comparing immune cell densities (CD3, CD4, CD45, CD45RO, FOXP3, and CD8) in *LHPP*-high and *LHPP*-low groups in the center of the tumor and invasive margin. Notably, higher densities of CD3+ and CD8+ cells are observed in *LHPP*-high samples, suggesting an association with increased immune cell infiltration. Statistical significance was determined using Student's t-tests. *, $P < 0.05$; **, $P < 0.01$; ***, $P < 0.001$.

Figure S18 (A) Immunohistochemistry (IHC) staining on tumour tissue microarrays (TMAs) was used to determine *LHPP* and *CD44* expression levels in 233 paraffin-embedded TMA specimens from our centre. Scale bar = 400 μm. **(B)** Overall survival curves for gastric cancer patients with low and high *LHPP* expression. **(C)** Overall survival curves for gastric cancer patients with low and high *CD44* expression. **(D)** Kaplan-Meier survival curves depicting overall survival probabilities for gastric cancer patients based on combined *LHPP* and *CD44* expression levels. Patients are classified into four groups: *LHPP*^{high} *CD44*^{high}, *LHPP*^{high} *CD44*^{low}, *LHPP*^{low} *CD44*^{high}, and *LHPP*^{low}

CD44^{low}.
