# Supplementary figures and images for: Targeting LHPP in neoadjuvant chemotherapy resistance of gastric cancer: insights from single-cell and multi-omics data on tumor immune microenvironment and stemness characteristics
Source: Cell Death Dis. 2025 Apr 16;16(1):306. doi: 10.1038/s41419-025-07614-z (PMC12003742; doi:10.1038/s41419-025-07614-z)

# Full length western blots

## Figure 5

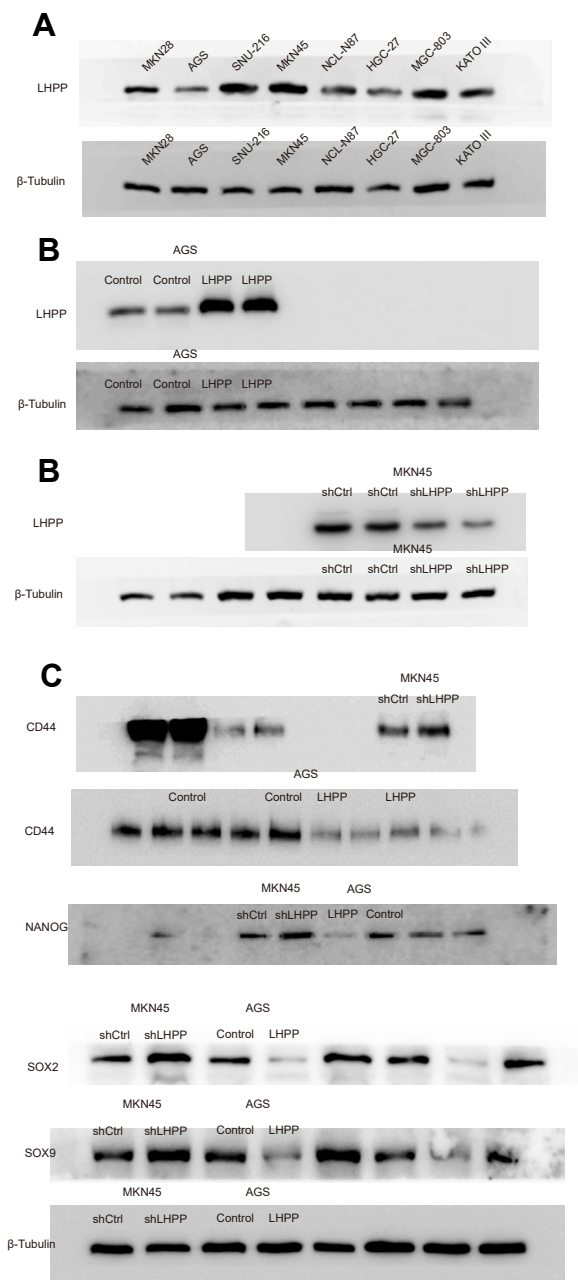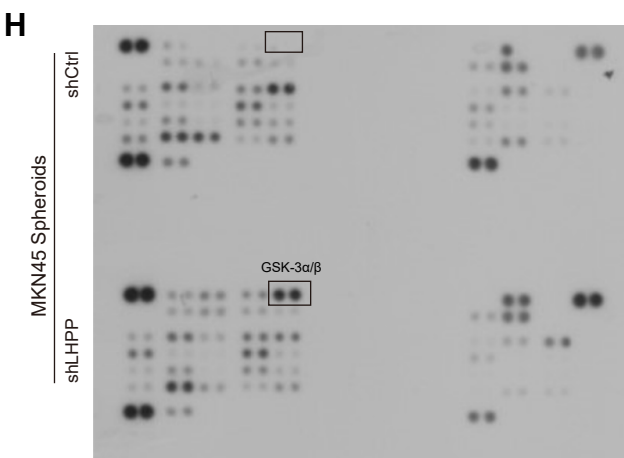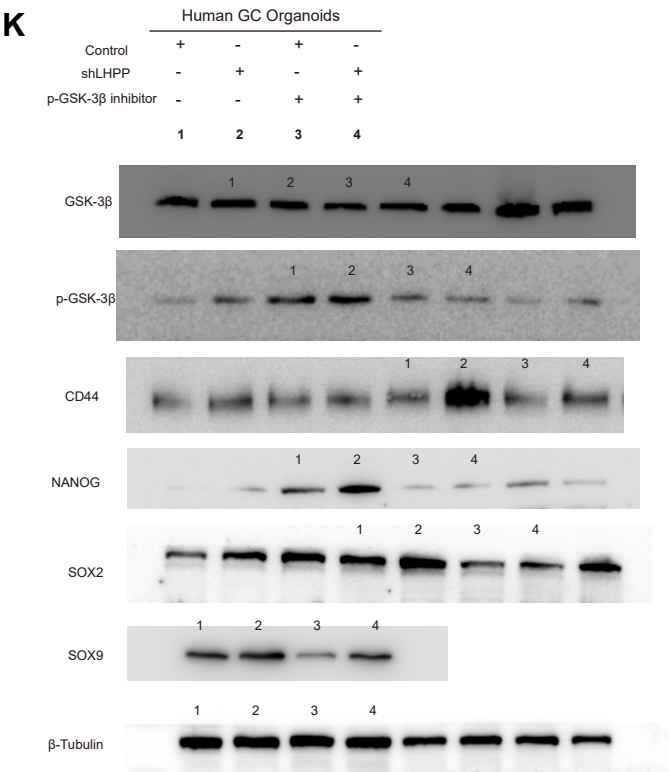

Figure S15

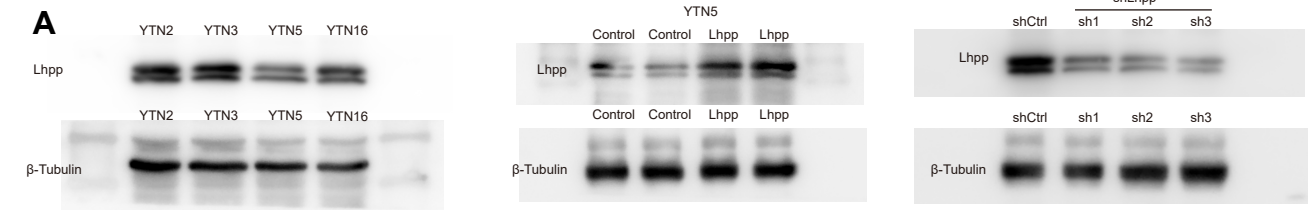

Supplement: Supplementary file 3 — Full length western blots [file 41419_2025_7614_MOESM3_ESM.pdf]
